# Organic Afterglow Vesicles

**DOI:** 10.1002/advs.202523635

**Published:** 2026-01-08

**Authors:** Siqi Zhu, Biao Xu, Ting Luo, Hui Li, Xinyi Wu, Siqi Zheng, Haodong Li, Yong Gao, Kaka Zhang

**Affiliations:** ^1^ Jiangsu Key Laboratory of Environmentally Friendly Polymeric Materials School of Materials Science and Engineering Jiangsu Collaborative Innovation Center of Photovoltaic Science and Engineering Changzhou University Changzhou P. R. China; ^2^ State Key Laboratory of Organometallic Chemistry and Shanghai Hongkong Joint Laboratory in Chemical Synthesis Key Laboratory of Synthetic and Self‐Assembly Chemistry for Organic Functional Molecules Ningbo Zhongke Creation Center of New Materials Shanghai Institute of Organic Chemistry University of Chinese Academy of Sciences Chinese Academy of Sciences Shanghai P. R. China; ^3^ Art and Science Research Center School of Humanities and Social Sciences University of Science and Technology of China Hefei Anhui P. R. China

**Keywords:** difluoroboron β‐diketonate, organic afterglow, polymerization‐induced self‐assembly, thermally activated delayed fluorescence, vesicles

## Abstract

Organic afterglow materials hold great promise for applications in bioimaging, sensing, and information encryption, yet the construction of advanced nanostructures like vesicles with long‐lived luminescence remains a formidable challenge. We pioneer the construction of organic afterglow vesicles via polymerization‐induced self‐assembly (PISA), integrating a thermally activated delayed fluorescence (TADF)‐type organic afterglow emitter into block copolymer nanostructures. The resulting vesicles exhibit well‐defined hollow morphologies, uniform size distribution, and high solid content up to 20%. They display significant room‐temperature afterglow with an emission lifetime exceeding 200 ms and a photoluminescence quantum yield (PLQY) of 20.8%. The afterglow mechanism is attributed to efficient TADF with a moderate intersystem crossing rate, where the triplet excited states are excellently protected by the rigid glassy vesicle walls. Moreover, the vesicles show rapid, reversible, and repeatable oxygen‐responsive behavior, making them promising for reusable oxygen sensing. This work establishes a versatile and scalable strategy for designing functional organic afterglow nanostructures with potential applications in bioimaging, sensing, and environmental monitoring.

## Graphical abstract

This work constructs organic afterglow vesicles through polymerization‐induced self‐assembly (PISA), integrating thermally activated delayed fluorescence (TADF) type organic afterglow emitters into block copolymer nanostructures. The resulting vesicles exhibited significant room‐temperature afterglow, with an emission lifetime exceeding 200 milliseconds and a photoluminescence quantum yield (PLQY) of 20.8%.

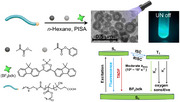

## 1 Introduction

Organic afterglow materials, owing to their long‐lived excited states, have demonstrated unique application potential in fields such as anti‐counterfeiting, information encryption, and hypoxic environment detection [1, 2, 3, 4, 5, 6, 7, 8, 9, 10]. The most common forms of organic afterglow materials include powders, bulk solids, single crystals, and polycrystalline aggregates. Some have been processed into thin films or panels, exhibiting intriguing properties for emerging flexible display and lighting technologies [11, 12, 13, 14, 15]. Another important but less explored form involves micro‐ and nanoscale afterglow particles dispersed in a medium. Notably, Fraser and co‐workers reported the use of room‐temperature phosphorescent polymer nanoparticles for oxygen mapping in cancerous tissues [16]. Pu and Huang developed water‐dispersible afterglow nanoparticles from luminescent small molecules for biomedical optical imaging, achieving high signal‐to‐noise ratios by leveraging their long‐lived emission in the afterglow imaging mode [17]. Li and colleagues further extended the emission wavelength of aqueous afterglow nanoparticles into the near‐infrared region and, in a specially designed system, achieved afterglow durations lasting up to tens of minutes [18]. Our group, along with several others, has also developed bioimaging‐capable afterglow micro/nanoparticles dispersed in aqueous or physiological media by employing organic matrix encapsulation and surfactant‐assisted strategies [19, 20, 21].

One commonly employed strategy for preparing dispersible micro/nanoscale afterglow particles is nanoprecipitation [22, 23, 24]. This method is operationally simple, does not require specialized equipment, and involves dissolving the luminescent molecules in a good organic solvent before injecting the solution into a poor solvent, typically in the presence of surfactants. During the precipitation process, micro/nanoparticles are formed as the luminescent molecular aggregate. However, due to the lack of highly‐ordered molecular packing during precipitation, the resulting particles often fail to establish a rigid microenvironment capable of effectively protecting the triplet excited states. Consequently, afterglow performance from nanoprecipitated particles is markedly inferior to that of the bulk solid‐state materials. Another approach is high‐power ultrasonication, which offers broader material compatibility [25, 26, 27]. In such method, bulk organic afterglow powders are sonicated in the presence of surfactants to produce micro/nanoparticles. However, the intense mechanical force of ultrasonication disrupts the rigid microenvironment that protects triplet states, and the high surface area of the resulting particles further increases susceptibility to quenching. As a result, afterglow properties are significantly compromised. To overcome the issue of afterglow performance decrease, our group developed an emulsion polymerization method [28, 29, 30, 31, 32]. In this strategy, organic luminescent molecules are directly incorporated into oil‐soluble monomers, which undergo free‐radical polymerization to form a rigid, glassy polymer matrix. This process leads to the in situ encapsulation of luminescent molecules within a protective polymer environment, without requiring harsh treatments such as ultrasonication that might damage the integrity of the triplet‐stabilizing microenvironment. As a result, the afterglow performance of the obtained micro/nanoparticles is comparable to that of the corresponding bulk state. Despite the merits of each aforementioned method, the resulting particle morphologies are typically limited to simple shapes such as solid spheres, rods, or irregular aggregates. To date, more complex structures such as vesicles or hollow spheres have not been successfully achieved in the context of afterglow‐active micro/nanomaterials.

Polymer vesicles (polymersomes), self‐assembled from amphiphilic block copolymers, exhibit a closed bilayer architecture analogous to that of liposomes, but with significantly enhanced physicochemical stability [33, 34, 35, 36]. Their unique structure—comprising a hydrophobic interlayer sandwiched between hydrophilic inner and outer surfaces—along with tunable properties such as size, permeability, and mechanical robustness, renders them highly valuable across diverse fields, including drug delivery, bioimaging and theranostics, biomimetic and membrane science, industrial and materials engineering, as well as environmental and energy applications [37, 38, 39, 40, 41]. To the best of our knowledge, polymer vesicles have never been integrated with organic afterglow systems. We envision that the successful combination of these two functional platforms could lead to multifunctional vesicles capable of simultaneously encapsulating therapeutic agents, imaging probes, and afterglow luminophores—thereby enabling integrated diagnosis and therapy. Such afterglow‐active vesicles could open new avenues in biomedical imaging, biosensing, in vitro diagnostics, biomimetic optical materials, and environmental monitoring. To realize this potential, a critical first step is the rational construction of organic afterglow vesicles.

**FIGURE 1:**
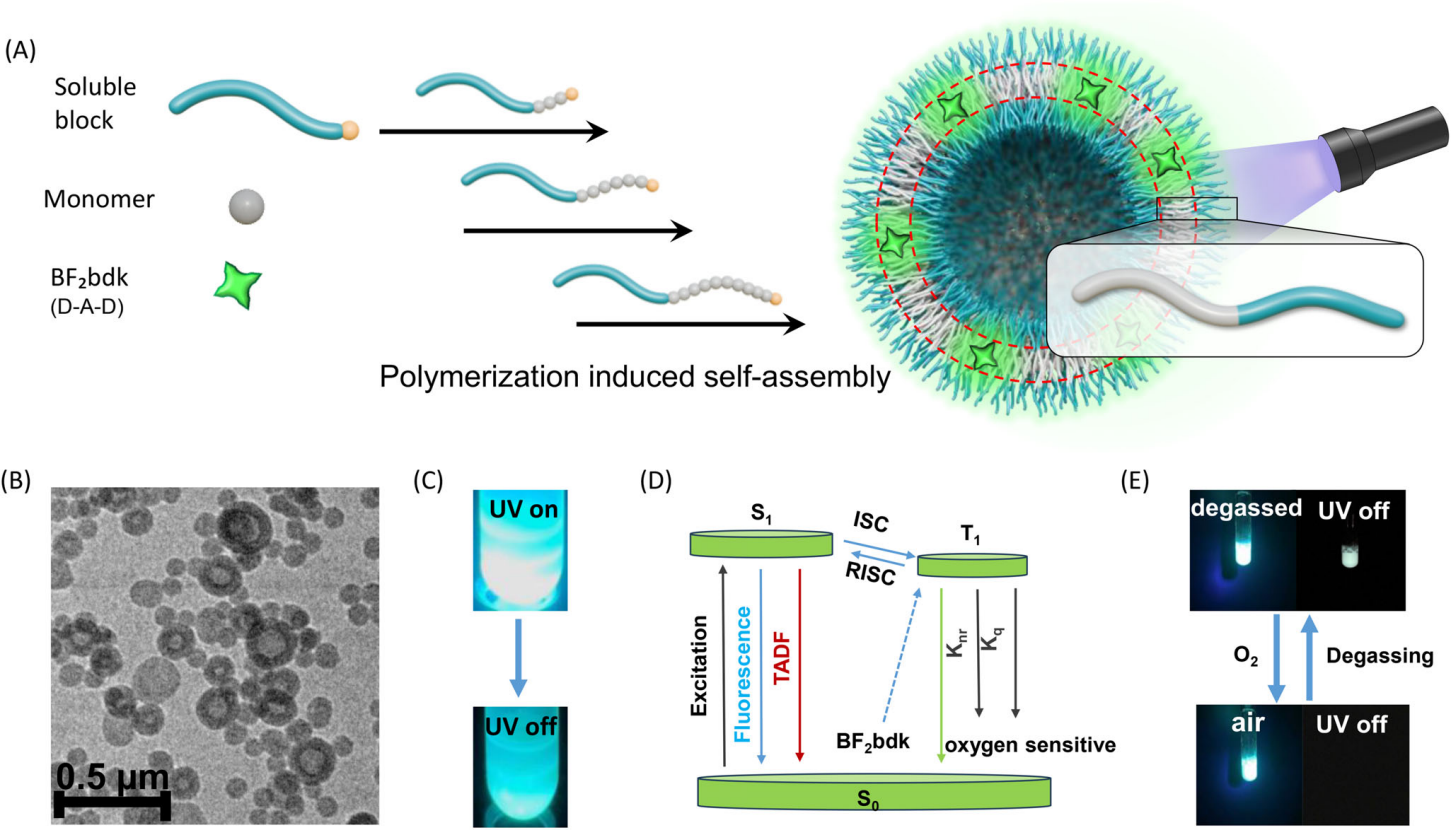
(A) Schematic illustration of the fabrication of organic afterglow vesicles by polymerization‐induced self‐assembly using TADF‐type BF_2_bdk emitters; (B) TEM images of the dried vesicles from the BF_2_bdk‐PISA dispersion; (C) Room‐temperature afterglow photographs of the BF_2_bdk‐PISA dispersion captured by iPhone14 cameras; (D) Perrin‐Jablonski diagram of the TADF‐type organic afterglow in the BF_2_bdk‐PISA dispersion; (E) Oxygen sensing function based on BF_2_bdk‐PISA dispersion.

Polymerization‐induced self‐assembly (PISA) is an advanced strategy that enables the in situ formation of amphiphilic block copolymer nanoassemblies—such as micelles, vesicles, and worm‐like structures—directly during polymerization [42, 43, 44, 45, 46, 47, 48, 49, 50, 51, 52, 53, 54, 55]. Unlike conventional self‐assembly, which requires pre‐synthesized block copolymers, PISA dynamically regulates the solvophobic–solvophilic balance throughout the polymerization process, allowing for the efficient one‐pot fabrication of functional nanostructures. The mechanism of PISA reveals a dynamic coupling between polymerization kinetics and evolving self‐assembled morphologies, and its scalability and high solids content make it particularly attractive for applications such as drug delivery systems and industrial coatings [43, 56, 57, 58, 59]. Here, we propose a PISA‐based strategy for the construction of organic afterglow vesicles. Inspired by our previous success with polymethyl methacrylate (PMMA)‐based afterglow emulsions, we selected difluoroboron β‐diketonate (BF_2_bdk) organic phosphors as the emissive components, in combination with PMMA or its copolymers as the solvophobic segment in the PISA formulation (Figure 1). Crucially, the solvophobic block (PMMA or its derivatives) not only serves to encapsulate and protect the triplet excited states of the afterglow luminophores after polymerization, but also possesses sufficient mobility during polymerization to drive the formation of vesicular structures (Figure 1A). The solvophilic block provides the resultant vesicles with excellent dispersibility in solvents. In this work, we successfully achieved the fabrication of organic afterglow vesicles via PISA (Figure 1B). The resulting vesicles exhibit prominent thermally activated delayed fluorescence (TADF)‐type afterglow behavior, with a lifetime longer than 200 ms and a photoluminescence quantum yield (PLQY) exceeding 20% (Figure 1C). These vesicles feature well‐defined morphology, uniform size distribution, and high solids content (Figure 1B). Moreover, they display fast and reversible response to molecular oxygen, allowing repeated activation cycles (Figure 1D). This study represents the first integration of a key synthetic polymer chemistry technique with organic room‐temperature afterglow systems, paving the way for a new class of functional luminescent materials.

## 2 Results and Discussion

**FIGURE 2:**
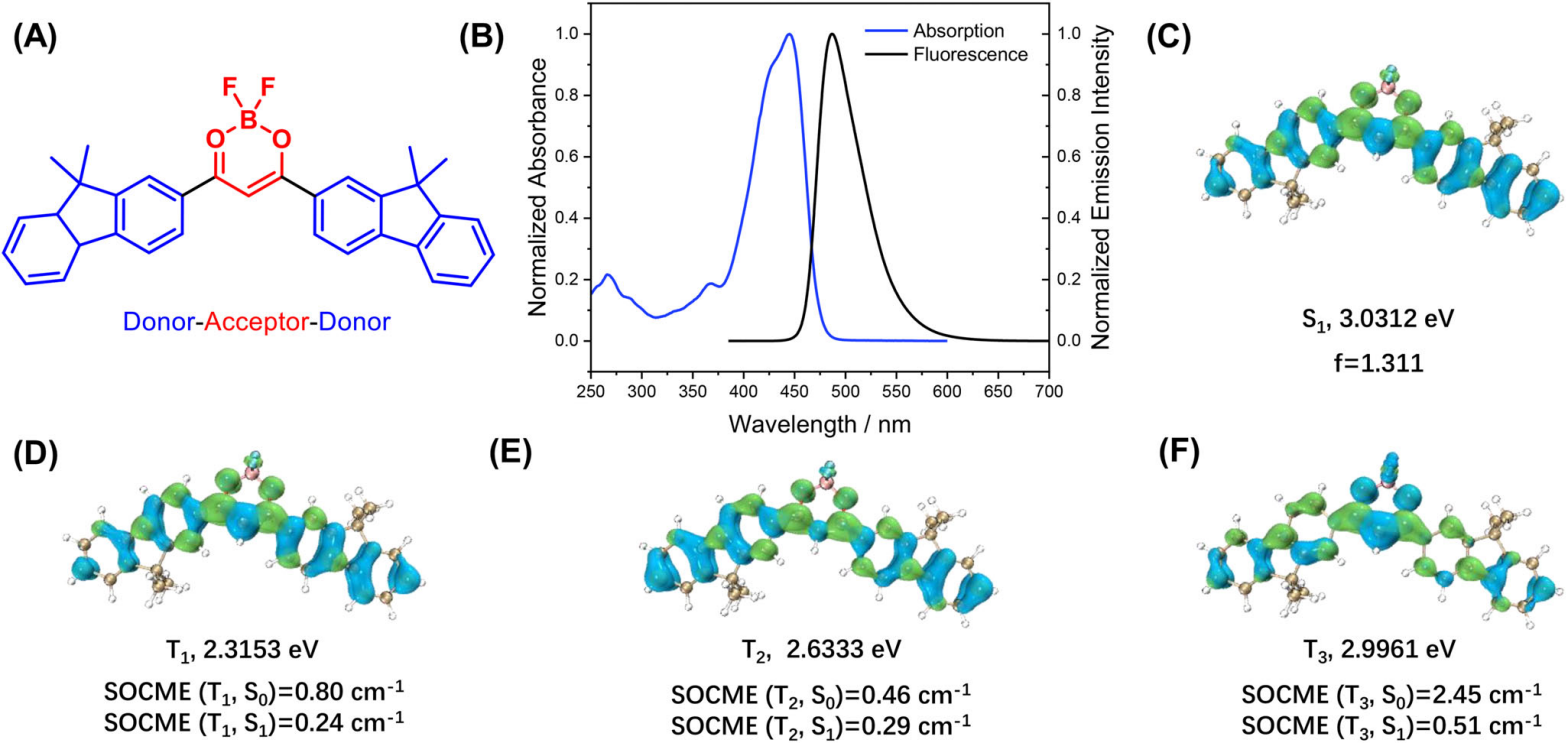
(A) Chemical structures of BF_2_bdk luminescent compounds; (B) UV–vis absorption spectrum and fluorescence spectrum of BF_2_bdk in dichloromethane at room temperature; (C‐F) Iso‐surface maps of electron‐hole density difference of BF_2_bdk's S_1_ (C), T_1_ (D), T_2_ (E) and T_3_ (F) excited states calculated at B3LYP/def2‐TZVP(‐f) level using optimized ground state geometry, where blue and green iso‐surfaces correspond to hole and electron distributions, respectively. SOCME values calculated on ORCA 4.2.1.

The BF_2_bdk molecule employed in this study adopts a donor–acceptor–donor (D–A–D) configuration, constructed from electron‐deficient difluoroboron (BF_2_) units and moderately electron‐donating 9,9‐dimethylfluorene groups (Figure 2A). This molecular design gives rise to a hybrid excited state with both locally excited (LE) and charge‐transfer (CT) character, facilitating multiple intersystem crossing (ISC) and reverse intersystem crossing (RISC) pathways. Time‐dependent density functional theory (TD‐DFT) calculations reveal that the spin–orbit coupling matrix elements (SOCME) between S_1_ and T_n_ (*n* = 1, 2, 3) are approximately 0.3 cm^−1^ or higher, indicating favorable spin‐flip processes (Figure 2C–F). According to the energy gap law and the El‐Sayed rule, such electronic configurations render BF_2_bdk an ideal candidate for organic afterglow systems, provided its triplet state (T_1_) can be effectively protected within a rigid matrix. BF_2_bdk exhibits a high molar absorption coefficient in dichloromethane, with a peak value of 7.14 × 10^4^ M^−1^ cm^−1^ at 445 nm (Figure 2B). Its fluorescence spectrum spans from 450 to 600 nm, with a maximum emission at 487 nm, and a photoluminescence quantum yield (PLQY) of 73.9% (Figure 2B). Table S1 summarizes the photophysical property of the BF_2_bdk solution in different solvents. The positive solvatochromicity also supports the intramolecular charge transfer property of the BF_2_bdk system (Figure S1), which agrees with the TD‐DFT calculation results (Figure 1C).

The solubility of BF_2_bdk in methyl methacrylate (MMA) is approximately 0.1 wt.%, and it can be well dispersed in poly(methyl methacrylate) (PMMA). To construct afterglow nanostructures via polymerization‐induced self‐assembly (Figure 1A), n‐hexane was selected as the continuous phase. Poly(lauryl methacrylate) (PLMA), which dissolves readily in n‐hexane due to its long alkyl chains, was used as the solvophilic macro‐chain transfer agent (macro‐CTA). Such macro‐CTA can be obtained in a controlled manner and with high isolation yield (Supporting Information). PLMA macro‐CTA with low degree of polymerization was designed, which would be conducive to fabricating polymeric nanostructures with low curvature such as vesicles. PMMA served as the core‐forming block. Owing to their poor solubility in n‐hexane, both BF_2_bdk and PMMA preferentially migrate and aggregate into the solvophobic core of the forming polymer nanoparticles. As the MMA monomer is progressively consumed during the polymerization, BF_2_bdk gradually transfers from the solvent phase into the growing PMMA‐rich core, enabling the co‐assembly of BF_2_bdk with PLMA‐*b*‐PMMA to form functional nanostructures. To induce morphological transformation and enhance structural complexity, phenyl methacrylate (PMA) monomer—soluble in n‐hexane, but whose polymer is not—was introduced at a tailored ratio, enabling the formation of more elaborate morphologies, such as nanofibers and vesicles (Figure 1).

**FIGURE 3:**
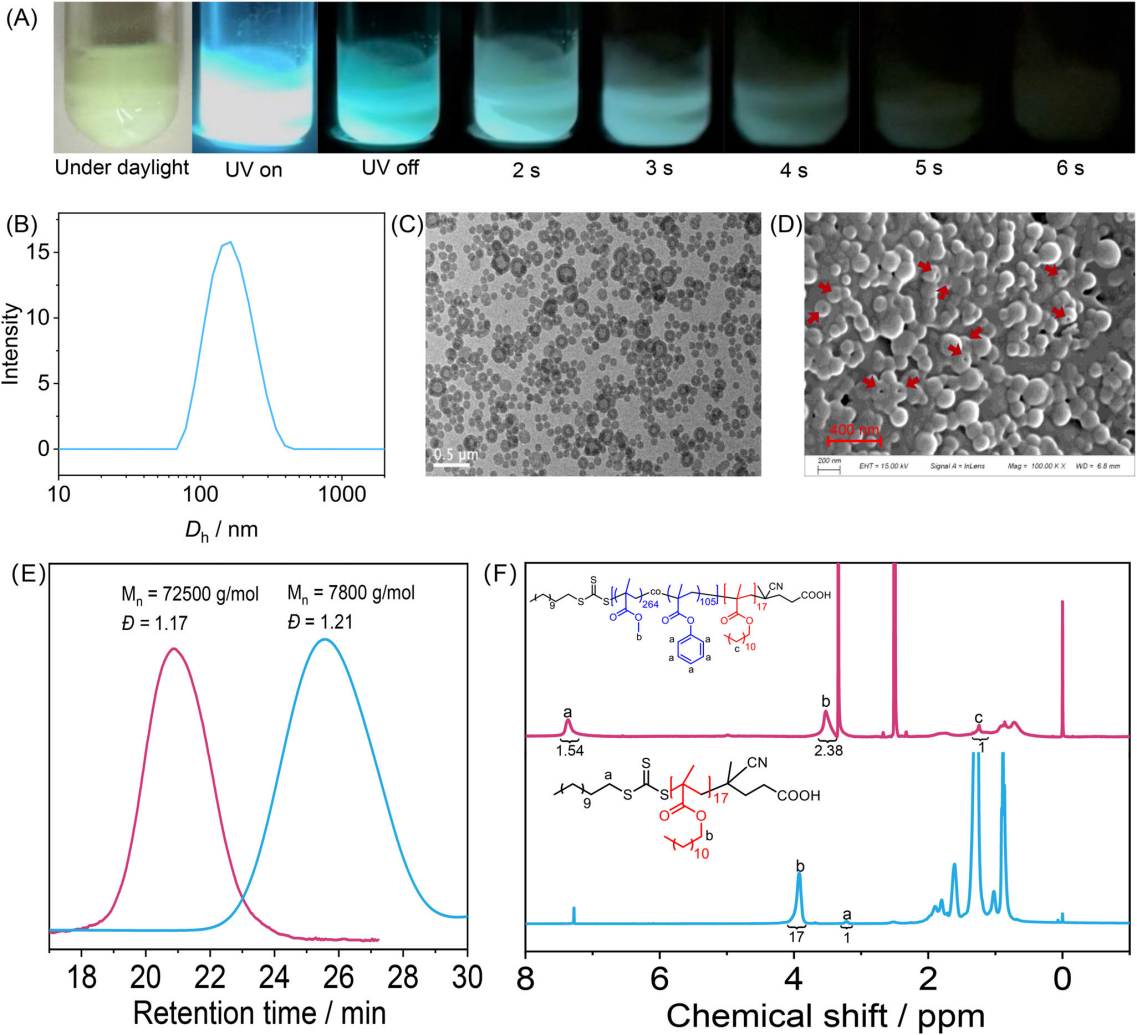
(A) Room‐temperature afterglow photographs of the BF_2_bdk‐PISA dispersion captured by iPhone14 cameras; (B) DLS trace of the BF_2_bdk‐PISA dispersion in n‐hexane; (C) TEM and (D) SEM images of the dried vesicles from the BF_2_bdk‐PISA dispersion; (E) GPC traces of PLMA_17_ and PLMA_17_‐*b*‐P(MMA_269_‐*co*‐PMA_105_) using tetrahydrofuran (THF) as the mobile phase; (F) ^1^H NMR spectra of PLMA_17_ and PLMA_17_‐*b*‐P(MMA_269_‐*co*‐PMA_105_).

In a representative PISA system formulated with PLMA_17_ as the macro‐CTA (the subscript represents degree of polymerization), a MMA/PMA feed molar ratio of 3:1, and a target degree of polymerization (DP) of 400, BF_2_bdk was introduced at a loading of 0.1 wt.% under oxygen‐free conditions. The polymerization was carried out at 70°C using azodiisobutyronitrile (AIBN) as the thermal initiator for 24 h, yielding a stable PISA dispersion composed of BF_2_bdk‐loaded PLMA_17_‐P(MMA_269_‐*co*‐PMA_104_) co‐assemblies with a solid content up to 20% (experimental procedures detailed in Supporting Information). Upon UV excitation at room temperature, the as‐prepared PISA dispersion exhibited bright blue‐green luminescence (Figure 3A). After ceasing UV irradiation, a persistent afterglow was clearly visible in a dark room, lasting for nearly 6 s (Figure 3A). Dynamic light scattering (DLS) analysis was also conducted to characterize the size distribution, showing an average hydrodynamic diameter (*D*_h_) of 255 nm and a polydispersity index of 0.016 (Figure 3B). Transmission electron microscope (TEM) observation of diluted samples in the dried state revealed polymer nanoparticles with vesicular morphologies filling the entire field of view (Figure 3C). These vesicles exhibited well‐defined morphology, with an average outer and inner diameter of 240 and 150 nm, respectively, corresponding to an average wall thickness of 45 nm (Figure 3C). Scanning electron microscopy (SEM) further confirmed the existence of abundant nanoscale polymer structures with dimensions consistent with TEM results. The presence of concave surface in SEM images, as indicated by the arrow, provided additional evidence supporting the hollow vesicular morphology of the PISA co‐assemblies (Figure 3D). ^1^H NMR analysis determined the degree of polymerization (DP) of the P(MMA‐*co*‐PMA) block to be 374 (Figure 3F), with an actual MMA/PMA composition of 0.72/0.28 (mol/mol). Gel permeation chromatography (GPC) traces of PLMA_17_ and PLMA_17_‐*b*‐P(MMA_269_‐*co*‐PMA_105_) exhibited a symmetrical unimodal peak with a quite narrow distribution. Relative to linear polystyrenes, the number‐averaged molecular weight (*M*_n_) of PLMA_17_ and PLMA_17_‐*b*‐P(MMA_269_‐*co*‐PMA_105_) were ∼7800 g/mol (*Ð* = 1.21), and ∼72500 g/mol (*Ð* = 1.17), respectively, as revealed in Figure 3E. Due to its low molecular weight and lack of heavy atoms, the BF_2_bdk molecule is not visible in conventional TEM imaging. Nonetheless, given its extremely poor solubility in n‐hexane, it is reasonable to infer that BF_2_bdk preferentially partitions into the vesicle wall formed by the P(MMA‐*co*‐PMA) block, rather than remaining in the solvent phase. Importantly, neither BF_2_bdk solutions nor BF_2_bdk solids exhibit afterglow at room temperature upon UV exposure. In solution, the lack of a rigid matrix prevents protection of the triplet excited state (T_1_), while in the solid state, dense molecular packing induces strong intermolecular coupling that rapidly quenches T_1_. Therefore, the room‐temperature afterglow observed in the PISA dispersion (Figure 3A) is attributed to BF_2_bdk molecules embedded within a rigid microenvironment—specifically, the glassy vesicle walls composed of P(MMA‐*co*‐PMA), which uniquely provide sufficient restriction to preserve the triplet excitons. The glass transition temperature (*T*_g_) of the bulk P(MMA‐*co*‐PMA) copolymer was measured to be ∼110°C (Figure S2). Although *T*_g_ is expected to decrease in the hexane‐dispersed state, it still offers adequate rigidity to suppress nonradiative deactivation of the BF_2_bdk triplet state at room temperature.

**FIGURE 4:**
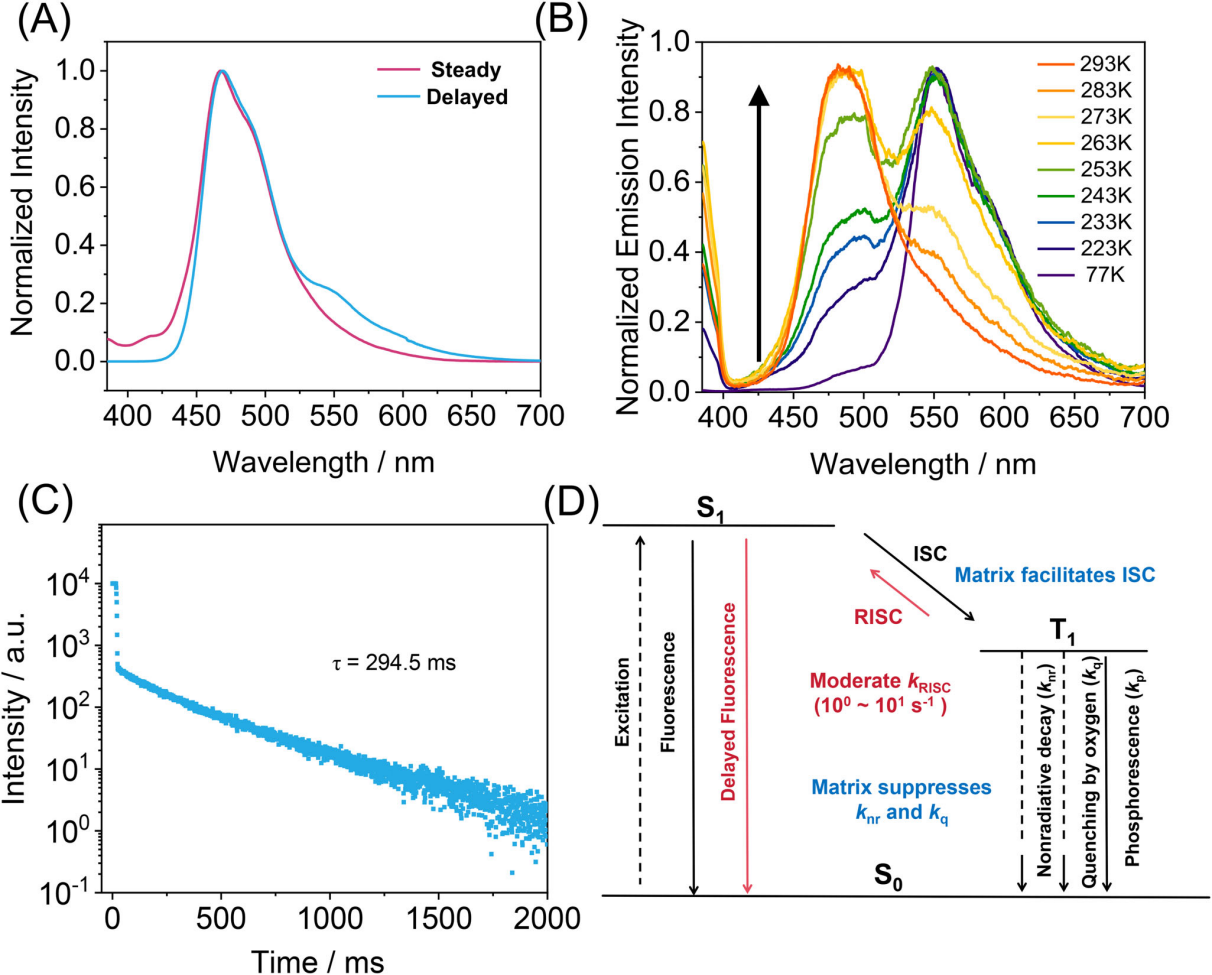
(A) Steady‐state and delayed emission spectra of the BF_2_bdk‐PISA dispersion at room temperature; (B) Temperature‐dependent delayed emission spectra (1 ms delay) of the BF_2_bdk‐PISA dispersion; (C) Room‐temperature emission decay of the BF_2_bdk‐PISA dispersion monitored at 475 nm; (D) Perrin‐Jablonski diagram of the TADF‐type organic afterglow in the BF_2_bdk‐PISA dispersion, featuring moderate *k*_RISC_ of 10^0^–10^1^ s^−1^ and excellent protection of organic triplet excited states by the glassy P(MMA‐*co*‐PMA) vesicle wall.

We next investigated the photophysical properties and underlying afterglow mechanism of the aforementioned BF_2_bdk‐PISA dispersion. At room temperature, steady‐state and delayed (1 ms delay) photoluminescence (PL) spectra of the BF_2_bdk‐PISA dispersion under 365 nm excitation were found to be nearly identical with fluorescence maxima at 475 nm (2.61 eV, Figure 4A). A similar spectral overlap was also observed under visible light excitation (Figure S3). Such spectral behavior is rarely reported and has only been documented in a few examples in the literature. One potential origin of overlapping steady‐state and delayed spectra is triplet‐to‐singlet energy transfer (TSET) from a long‐lived donor to a fluorescent acceptor [60]. However, this mechanism requires efficient excitation of the donor component. In our system, control PISA dispersions devoid of BF_2_bdk exhibit negligible absorption in both the UV and visible regions and show no afterglow under 365 nm irradiation, ruling out TSET as the dominant mechanism (Figure S4). Another plausible explanation lies in organic long‐persistent luminescence (OLPL), where donor–acceptor systems with intermolecular charge transfer and slow recombination kinetics can exhibit nearly identical steady and delayed emission spectra [7]. However, the P(MMA‐*co*‐PMA) matrix possesses a significantly lower HOMO and higher LUMO energy level compared to the BF_2_bdk molecule, making efficient intermolecular charge transfer between the matrix and BF_2_bdk highly unlikely. We also considered the possibility of impurity‐induced afterglow [61], which could yield overlapping emission profiles. This hypothesis was dismissed based on the rigorous purification of BF_2_bdk via column chromatography and recrystallization. High‐performance liquid chromatography (HPLC) analysis confirmed its high chemical purity (Figure S5). Further supporting this conclusion, the excitation spectrum of the BF_2_bdk‐PISA dispersion exhibited peak positions and line shapes nearly identical to the UV–vis absorption spectrum of the isolated BF_2_bdk dopant (Figure S6). While UV–vis absorption reflects the intrinsic absorption properties of all species in the system, the excitation spectrum is representative only of those species contributing to emission. Therefore, the strong spectral agreement between excitation and absorption spectra confirms that the BF_2_bdk dopant is the sole emissive species in the afterglow system, effectively excluding the involvement of unknown emissive impurities.

To gain deeper insights into the afterglow mechanism, we performed temperature‐dependent delayed emission spectroscopy on the BF_2_bdk‐PISA system. At 77 K, the PISA dispersion exhibited distinct yellow colored afterglow following cessation of 365 nm excitation (Figure S7). The delayed emission spectrum (1 ms delay) recorded under these conditions revealed a pronounced phosphorescence band in the range of 500–700 nm, with a peak at 552 nm (2.25 eV) (Figure 4B), which is attributable to the phosphorescent decay from the triplet excited state of the BF_2_bdk molecule. The singlet–triplet energy gap (Δ*E*_ST_) was estimated to be 0.36 eV, based on the peak positions of fluorescence and phosphorescence bands. Upon increasing the temperature, a progressive enhancement of delayed emission within the 400–550 nm range was observed, eventually dominating the entire delayed emission spectrum above 293 K (Figure 4B). This thermally activated behavior is indicative of a thermally activated delayed fluorescence (TADF) mechanism in the system. Delayed fluorescence can originate from either triplet–triplet annihilation (TTA) or TADF processes. However, given that TTA is a bimolecular process, its contribution becomes negligible at the low dopant concentration employed in this study (0.1 wt.%). Thus, the observed temperature‐dependent delayed emission is primarily attributed to TADF, rather than TTA.

Under ambient conditions, the excited‐state decay profile of the BF_2_bdk‐PISA system monitored at its emission maximum (475 nm) revealed a prolonged lifetime of 294.5 ms (Figure 4C). This lifetime is attributed to the delayed fluorescence (DF) component in a thermally activated delayed fluorescence (TADF) system, arising from a reverse intersystem crossing (RISC) process from the triplet (T_1_) to singlet (S_1_) excited state (Figure 4D). Additionally, time‐resolved measurements at 450 nm showed a similarly long decay exceeding 100 ms at room temperature (Figure S8). Notably, the 77 K delayed emission spectrum (Figure 4B) displayed no phosphorescent contribution at this wavelength, affirming that the long‐lived emission at 450 nm under ambient conditions stems from TADF rather than phosphorescence.

The vesicular system exhibited a high photoluminescence quantum yield (PLQY) of 20.8% at room temperature. Based on the room‐temperature delayed fluorescence lifetime of 294.5 ms, the RISC rate constant (*k*_RISC_) was estimated to lie in the range of 10^0^–10^1^ s^−1^ at room temperature. Given that RISC is the rate‐limiting step in TADF‐based afterglow systems, the overall afterglow lifetime (τ_AG_) can be expressed as: τ_AG_ = 1 / (*k*_RISC_ + *k*_P_ + *k*_nr_(T_1_) + *k*_q_(T_1_)) where *k*_P_ is the radiative decay rate from T_1_, *k*_nr_ is the nonradiative decay rate, and *k*_q_ accounts for oxygen quenching of the triplet state. Phosphorescence lifetime measurements of BF_2_bdk at 77 K revealed a value of 1662 ms (Figure S9), where both *k*_RISC_ and *k*_q_(T_1_) can be neglected and *k*_nr_(T_1_) is minimized due to the frozen matrix. From these data, the *k*_P_ value was estimated to be 0.6 s^−1^. In the glassy matrix formed by P(MMA‐*co*‐PMA), triplet‐state nonradiative decay *k*_nr_(T_1_) is substantially suppressed at room temperature. Applying the relation *k*_RISC_ = 1/τ_AG_ – *k*_P_, we obtained a *k*_RISC_ value of 2.8 s^−1^ for the BF_2_bdk‐PISA dispersion at room temperature. At room temperature, the *k*_nr_(T_1_) can be suppressed to very small values because the luminophores are embedded in the glassy vesicle walls. Besides, under anaerobic conditions of the PISA system, *k*_q_(T_1_) can be minimized. As a result, the moderate yet clearly dominant *k*_RISC_ (≫ *k*_P_) effectively enables the TADF afterglow mechanism, facilitating triplet exciton utilization while maintaining an emission lifetime over 0.1 s—crucial for achieving efficient persistent luminescence. It is noteworthy that the *k*_RISC_ value in this system is significantly lower than those of conventional TADF emitters used in OLEDs, which typically fall within the 10^3^–10^6^ s^−1^ range [62]. Such a difference stems from the distinct molecular design criteria required for afterglow versus OLED emitters. Previous studies have established that achieving *k*_RISC_ rates on the order of 10^3^–10^6^ s^−1^ generally necessitates (1) a small singlet–triplet energy gap (Δ*E*_ST_ < 0.2 eV), and (2) a large spin–orbit coupling matrix element (SOCME > 0.3–0.5 cm^−1^) [62, 63]. In our system, TD‐DFT calculations revealed SOCME values of 0.24, 0.29, and 0.51 cm^−1^ for the S_1_–T_1_, S_1_–T_2_, and S_1_–T_3_ transitions, respectively (Figure 2D–F). The Δ*E*_ST_ of the BF_2_bdk‐PISA system was determined to be 0.36 eV. According to literature reports, an increase of 0.1 eV in Δ*E*_ST_ leads to an approximate 100‐fold decrease in *k*_RISC_ [63]. Thus, a Δ*E*_ST_ around 0.3–0.4 eV supports *k*_RISC_ values in the range of 10^0^–10^1^ s^−1^, consistent with our experimental findings and prior studies. Importantly, the glassy matrix of P(MMA‐*co*‐PMA) helps preserve the long‐lived triplet excitons by minimizing nonradiative decay (*k*_nr_), while the deoxygenated environment ensures that the oxygen quenching constant (*k*_q_) remains negligible. These combined effects enable the emergence of efficient TADF‐based organic afterglow in the vesicular nanostructures (Figure 4D).

To elucidate the formation mechanism of the organic afterglow vesicles, a systematic series of PISA reactions was conducted by varying both the target degree of polymerization (DP) and the feed molar ratio of MMA to PMA (Table 1). With a fixed MMA/PMA molar ratio of 3:1, we explored target DPs of 200, 400, and 500. Based on ^1^H NMR analysis, the monomer conversions were ∼95%, ∼94%, and ∼83% after 24 h of polymerization, respectively. A clear morphological evolution was observed—from 2D fiber to well‐defined polymeric vesicles and ultimately to vesicle aggregates (Figure S10). This transformation is attributed to a gradual increase of the packing parameter, *p*, as the relative length of the P(MMA‐*co*‐PMA) block increases compared to the PLMA shell‐forming segment. At a feed ratio of MMA/PMA  =  4:1 (i.e., 8/2), cylindrical micelles with varying degrees of branching were consistently observed across actual DP  =  94, 199, and 465 (Figure S11). The cylinder diameter increased with higher DP, consistent with polymer chain growth. When the MMA/PMA ratio was further increased to 9:1, wormlike micelles with well‐defined contours were formed at actual DP  =  96 and 180 (Figure S12). In contrast, when no PMA was introduced into the formulation, the PISA products at DP  =  200 and 400 exhibited irregular composite structures composed of fiber‐like and spherical domains (Figure S13). These disordered morphologies are likely a consequence of the limited segmental mobility of PMMA in n‐hexane, which hinders morphology evolution and traps the system in kinetically favored, metastable states. Despite their distinct nanostructures, all of the above assemblies displayed robust room‐temperature afterglow emission (Figure S14). Representative sample including PLMA_17_‐*b*‐P(MMA_80_‐*co*‐PMA_20_) was selected for detailed photophysical characterization. In each case, the steady‐state and delayed emission spectra at room temperature were nearly identical, and temperature‐dependent measurements revealed a progressive enhancement of delayed fluorescence with increasing temperature (Figure S15). These observations confirm that all of the examined morphologies exhibit room‐temperature TADF‐type afterglow behavior (Table 1). Table 1 shows that the molecular weight (M_n_) from GPC are much higher than that from NMR. For the NMR method, we first calculate the average degree of polymerization for each block from the integral ratios in the ^1^H NMR spectra, and then the M_n_ can be readily obtained by assuming all the polymer chains in the sample follow the PLMA‐*b*‐P(MMA‐*co*‐PMA) structure. In actuality, during PISA, there would be coupling termination, especially in the case of high monomer conversion. The resultant polymer chains after coupling termination have much higher molecular weights which cannot be distinguished by NMR method but can be revealed by GPC method (in GPC, polymers are separated according to their molecular weights or hydrodynamic volume). The occurrence of coupling termination should be the main reason for the observation that the molecular weight (M_n_) from GPC are much higher than that from NMR as shown in Table 1.

**TABLE 1:** Experimental results of BF_2_bdk‐PISA dispersion obtained from different reaction condition.

Sample	Con. (%)	PMA/MMA (molar ratio)		M_n, GPC_ (×10^3^)	M_n, NMR_	*Ð*	Morph.	After glow (s)
		in feed	in copolymer					
PLMA_17_‐*b*‐PMMA_190_	95.5	0:1	0:1	97	24225	1.21	fiber	9
PLMA_17_‐*b*‐PMMA_380_	95	0:1	0:1	118.8	43247	1.24	fiber	7
PLMA_17_‐*b*‐P(MMA_84_‐*co*‐PMA_12_)	96	1:9	1.3:8.7	32.2	14744	1.07	worm	7
PLMA_17_‐*b*‐P(MMA_160_‐*co*‐PMA_20_)	90	1:9	1.1:8.9	52	23123	1.13	worm	7
PLMA_17_‐*b*‐P(MMA_433_‐*co*‐PMA_48_)	96.2	1:9	1:9	155.6	53258	1.57	fiber	7
PLMA_17_‐*b*‐P(MMA_75_‐*co*‐PMA_19_)	94	2:8	2:8	27.6	15259	1.16	worm	6
PLMA_17_‐*b*‐P(MMA_173_‐*co*‐PMA_26_)	99	2:8	1.3:8.7	65	26631	1.22	worm	6
PLMA_17_‐*b*‐P(MMA_409_‐*co*‐PMA_56_)	93	2:8	1.2:8.8	146	55120	1.34	worm	7
PLMA_17_‐*b*‐P(MMA_134_‐*co*‐PMA_55_)	94.5	1:3	2.9:7.1	26.8	27427	1.16	fiber	6
PLMA_17_‐*b*‐P(MMA_269_‐*co*‐PMA_105_)	93.5	1:3	2.8:7.2	72.5	49046	1.17	vesicle	6
PLMA_17_‐*b*‐P(MMA_295_‐co‐PMA_121_)	83.2	1:3	2.9:7.1	102.4	54239	1.41	vesicle	6

**FIGURE 5:**
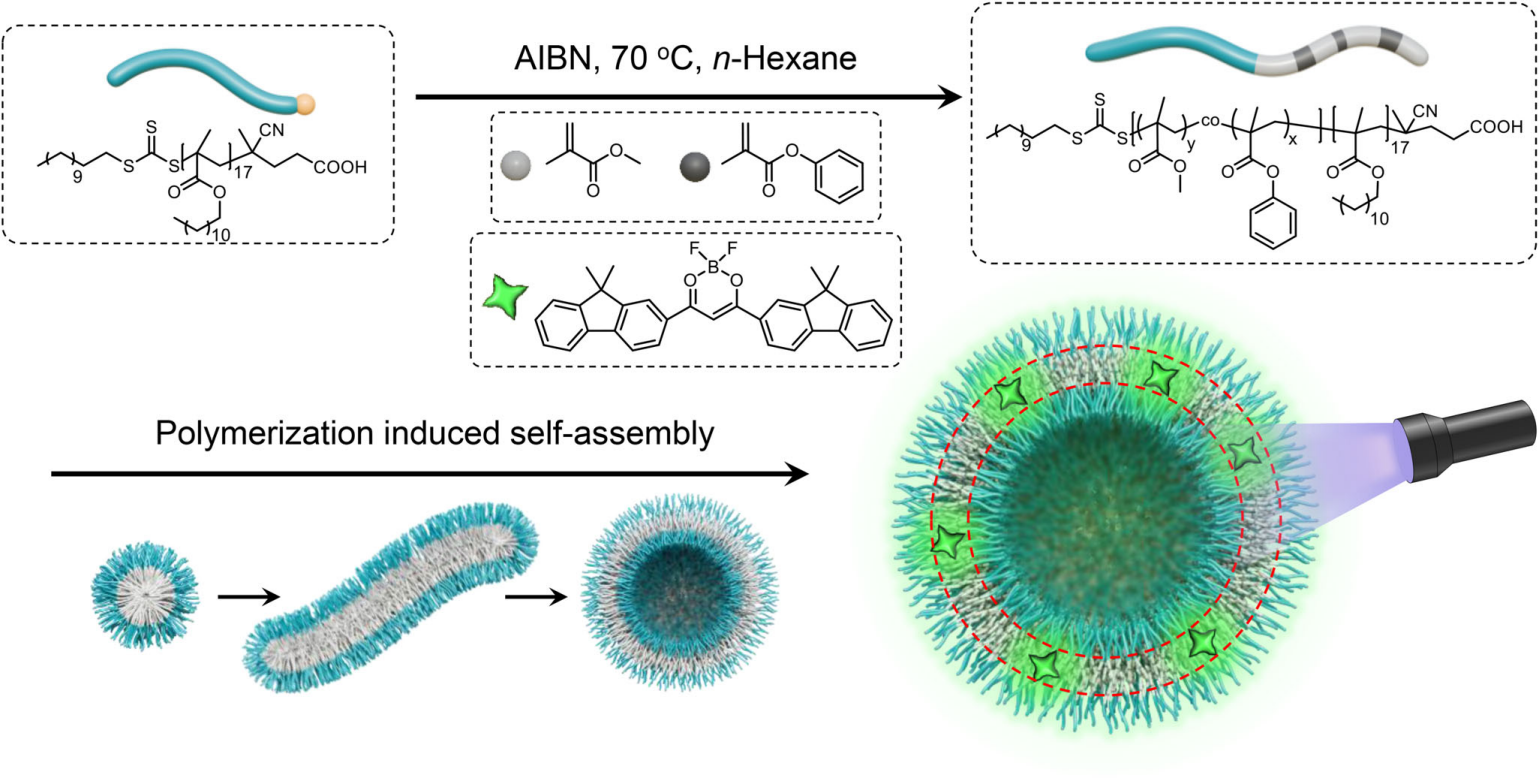
Schematic illustration of the fabrication of organic afterglow vesicles by polymerization‐induced self‐assembly using TADF‐type BF_2_bdk emitters.

Based on the above findings, we propose a mechanistic model for the formation of organic afterglow vesicles (Figure 5). Prior to PISA, the BF_2_bdk is molecularly dissolved in the mixture of MMA, PMA and n‐hexane. Upon thermal initiation at 70 °C under oxygen‐free conditions, AIBN decomposes to generate radicals, triggering controlled radical polymerization governed by the PLMA chain transfer agent (macro‐CTA). As the amphiphilic block copolymer PLMA‐*b*‐P(MMA‐*co*‐PMA) grows, the insoluble P(MMA‐*co*‐PMA) block begins to aggregate, forming the core of the self‐assembled structures, while the soluble PLMA block stabilizes the assemblies in the nonpolar solvent. During polymerization, MMA and PMA monomers are gradually consumed, and the solubility of BF_2_bdk in the diminishing solvent phase decreases. Consequently, BF_2_bdk preferentially partitions into the growing hydrophobic P(MMA‐*co*‐PMA) core domains (Figure 5). Since short PLMA was used as shell‐forming chains, the increase of P(MMA‐*co*‐PMA) core‐forming chains would give a large length ratio of P(MMA‐*co*‐PMA)/PLMA chain. Block copolymers with such structural parameter would assemble into polymeric vesicles with low curvature. Besides, in the PISA system with a MMA/PMA molar ratio of 3:1, the presence of PMA and formation of the P(MMA‐*co*‐PMA) block would facilitate the system to undergo morphology transitions and evolve into well‐defined vesicular nanostructures as polymerization proceeds. Upon completion of polymerization and cooling to room temperature, the BF_2_bdk molecules become confined within the rigid glassy matrix of the P(MMA‐*co*‐PMA) vesicle walls. Under oxygen‐free conditions, excitation by UV or visible light promotes the BF_2_bdk molecules to their singlet excited state (S_1_), which subsequently undergoes intersystem crossing (ISC) to various triplet states (T_1_, T_2_, etc.) facilitated by the energy gap law and the El‐Sayed rule. The rigid glassy microenvironment and the absence of oxygen jointly ensure effective protection of the triplet excitons, significantly suppressing nonradiative decay. In this matrix, the phosphorescent decay rate (*k*_P_) is about 0.6 s^−^^1^, while the moderate RISC rate constant (*k*_RISC_ ≈ 10^0^–10^1^ s^−1^) is sufficient to facilitate efficient reverse intersystem crossing and enable TADF‐type afterglow. This design thus leverages a polymeric confinement strategy to protect the organic triplet emitters and realize long‐lived luminescence (Figure 5).

**FIGURE 6:**
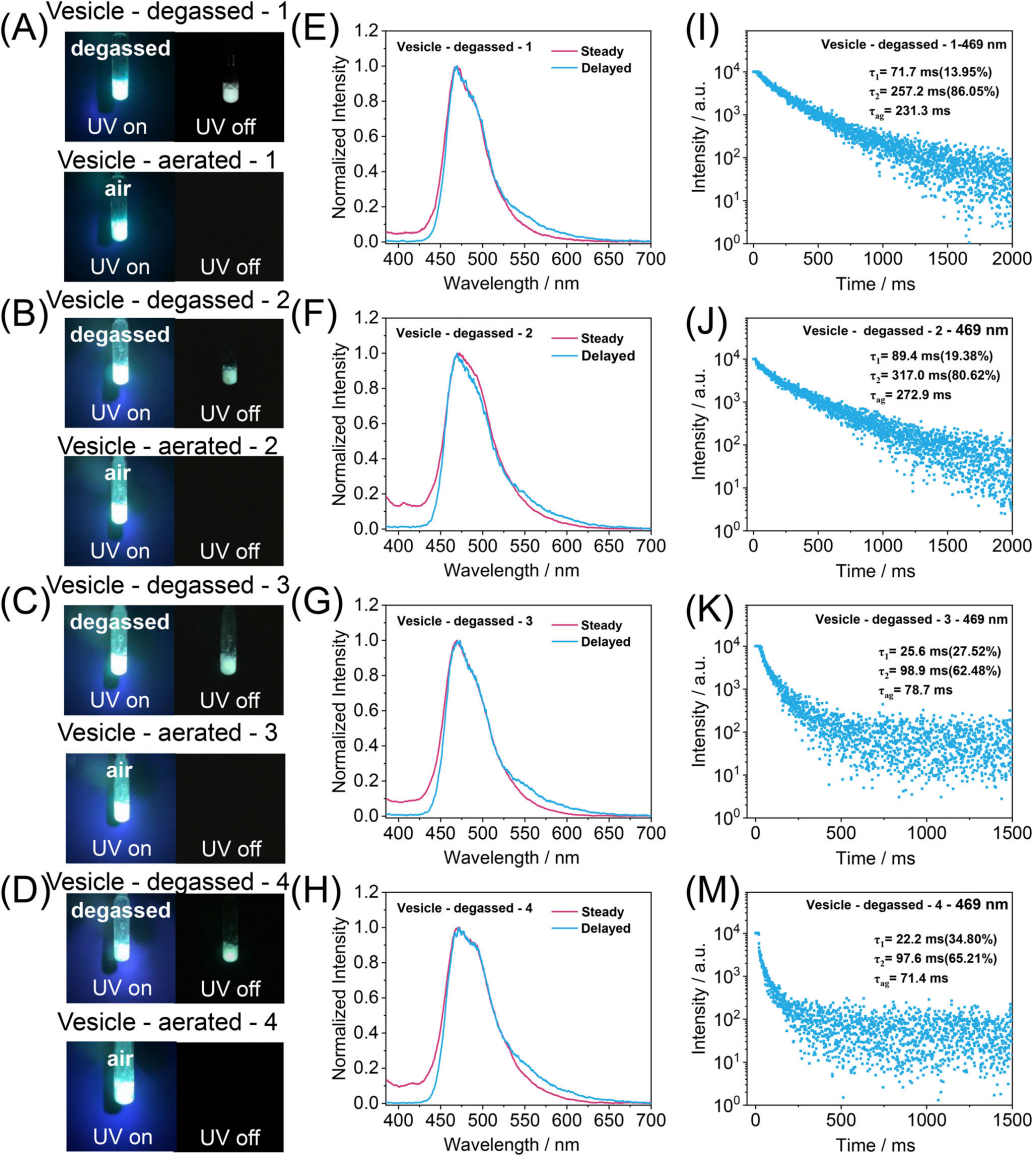
(A‐D) Photographs of BF_2_bdk‐PISA afterglow vesicles under a 365 nm UV lamp and after removal of the UV lamp at degassed and aerated conditions. (E‐H) Room‐temperature steady‐state and delayed spectra (1 ms delay) of BF_2_bdk‐PISA afterglow vesicles under degassed condition. (I‐M) Room‐temperature emission decay of BF_2_bdk‐PISA afterglow vesicles afterglow emulsion under degassed condition.

By integrating the BF_2_bdk luminophore into a polymerization‐induced self‐assembly platform, we report, for the first time, the controllable construction of organic afterglow vesicles—a nanostructure that has not been previously documented in the literature. These vesicles exhibit high solid content, well‐defined morphology, and uniform size distribution. Owing to the intrinsic oxygen permeability of the P(MMA‐*co*‐PMA) wall, the vesicles display distinct oxygen responsiveness. Upon exposure to ambient air, the afterglow emission rapidly vanishes (Figure 6A), which can be attributed to both the high oxygen diffusivity of P(MMA‐*co*‐PMA) vesicle wall and the nanoscale dimensions of the vesicles, characterized by large surface‐to‐volume ratios and thin walls (45 nm). Oxygen molecules readily penetrate the vesicle membrane and quench the triplet states of the encapsulated BF_2_bdk emitters, effectively shutting off the TADF‐type afterglow. Notably, this quenching process is fully reversible: upon degassing, the room‐temperature afterglow recovers, and the vesicles consistently demonstrate repeatable on–off switching behavior in response to oxygen cycling (Figure 6). These features make the organic afterglow vesicles excellent candidates for reusable oxygen‐sensing platforms. More broadly, our study establishes a scalable and modular strategy for constructing advanced organic afterglow nanostructures by leveraging the versatility of the PISA approach. Further, we use other luminophores such as donor‐acceptor type BF_2_bdk, coronene and 10‐phenylacridone for PISA, and the resultant PISA dispersion exhibit room‐temperature phosphorescence, very long phosphorescence lifetime of 4 s and blue afterglow, respectively (Figures S16–S18). These studies enrich the chemical compositions and afterglow properties of the present system. The successful preparation of the afterglow PISA dispersion requires (1) the excellent afterglow property of the luminophores and (2) the solubility matching of the luminophores and the core‐forming blocks. Here the BF_2_bdk, coronene and 10‐phenylacridone are insoluble in n‐hexane but soluble in the MMA/n‐hexane solvent mixture before PISA. The luminophores would become insoluble during polymerization because of the decrease of MMA ratio in the solvent mixture, and consequently the luminophores and the core‐forming blocks co‐assemble into the polymeric nanostructures. The resulting materials combine long‐lived luminescence, morphological tunability, and environmental responsiveness, offering a new class of multifunctional soft‐matter phosphors with promising potential in sensing, imaging, and beyond.

## 3 Conclusion

In summary, we have developed a novel and robust PISA‐based strategy to fabricate organic afterglow vesicles—a previously unreported nanostructure in the field of organic afterglow materials. These vesicles combine the advantages of polymeric self‐assembly and TADF‐type afterglow, featuring well‐defined hollow morphologies, high colloidal stability, and long‐lived emission at room temperature. The incorporation of BF_2_bdk luminophores into the rigid P(MMA‐*co*‐PMA) matrix effectively protects triplet excitons, enabling efficient TADF with a lifetime over 200 ms. The system also exhibits reversible oxygen‐responsive behavior, allowing for repeated on–off switching of the afterglow, which highlights its potential as a reusable oxygen sensor. This study not only introduces a new class of organic afterglow nanomaterials but also demonstrates the power of PISA as a modular and scalable platform for constructing advanced functional luminescent materials. We anticipate that this approach will inspire further exploration of multifunctional afterglow systems for a wide range of applications in biomedicine, optoelectronics, and smart materials.

## Conflicts of Interest

The authors declare no conflicts of interest.

## Supporting information




**Supporting File**: advs73742‐sup‐0001‐SuppMat.pdf.

## Data Availability

The data that support the findings of this study are available from the corresponding author upon reasonable request.
