# Supplementary material for: Organic Afterglow Vesicles
Source: Adv Sci (Weinh). 2026 Jan 8;13(16):e23635. doi: 10.1002/advs.202523635 (PMC13042688; doi:10.1002/advs.202523635)
Supplement: Supplementary file 1 — Supporting File: advs73742‐sup‐0001‐SuppMat.pdf. [file ADVS-13-e23635-s001.pdf]

# Supporting Information for

# Organic Afterglow Vesicles

*Siqi Zhu, # [1] Biao Xu, # [2] Ting Luo, [2] Hui Li, [1] Xinyi Wu, [1] Siqi Zheng, [1] Haodong Li, [3] Yong Gao, *[1] Kaka Zhang *[2]*

[1] Jiangsu Key Laboratory of Environmentally Friendly Polymeric Materials, School of Materials Science and Engineering, Jiangsu Collaborative Innovation Center of Photovoltaic Science and Engineering, Changzhou University, 21 Gehuzhong Road, Changzhou 213100, People's Republic of China. E-mail: gydx.1027@163.com

[2] State Key Laboratory of Organometallic Chemistry and Shanghai Hongkong Joint Laboratory in Chemical Synthesis, Key Laboratory of Synthetic and Self-Assembly Chemistry for Organic Functional Molecules, Ningbo Zhongke Creation Center of New Materials, Shanghai Institute of Organic Chemistry, University of Chinese Academy of Sciences, Chinese Academy of Sciences, 345 Lingling Road, Shanghai 200032, People's Republic of China. E-mail: zhangkaka@sioc.ac.cn

[3] Art and Science Research Center, School of Humanities and Social Sciences, University of Science and Technology of China, No.96, Jinzhai Road, Baohe District, Hefei, Anhui, 230026, People's Republic of China.

#equal contribution

## Experimental Section

## Materials

Lauryl methacrylate (LMA, 98.3%), methyl methacrylate (MMA, 98%), and phenyl methacrylate (PMA, 99.95%) were purchased from Bide Pharmatech Ltd. (Shanghai, China). These vinyl monomers were separately purified by passing through a basic $Al_2O_3$ column prior to use. 2,2'-Azobisisobutyronitrile (AIBN) was purchased from Kemiou (Tianjin, China), which was recrystallized from ethanol before use and stored at low temperature. 4-Cyano-4-(dodecylthiothiocarbonylthio) pentanoic acid (CDPA) was synthesized in laboratory according to the procedures described elsewhere.[1] Diethyl ether and toluene were purchased from Sinopharm Chemical Reagent Co., Ltd. (Shanghai, China).

## Synthesis of Difluoroboron β-Diketonate ($BF_2$bdk)

Under a nitrogen atmosphere at 0°C, a reaction flask was charged with anhydrous aluminum chloride (266.7 mg, 2.0 mmol) and dichloromethane (10 mL), followed by the addition of 9,9-dimethylfluorene (233.0 mg, 1.2 mmol). After stirring for 10 minutes, malonyl chloride (70.5 mg, 0.5 mmol) was added dropwise, and the mixture was stirred for an additional 20 minutes. The reaction temperature was then raised to room temperature and stirring was continued for 8 hours. Upon completion, the reaction mixture was slowly poured into cooled 1 M HCl solution. The organic phase was extracted with dichloromethane, washed with deionized water and saturated sodium chloride solution, dried over anhydrous sodium sulfate, and concentrated under reduced pressure to afford the intermediate as a solid. Under a nitrogen atmosphere, the intermediate was dissolved in dichloromethane (10 mL), and boron trifluoride diethyl etherate (283.9 mg, 2.0 mmol) was added dropwise. The reaction was stirred at room temperature overnight. After completion, the mixture was quenched with deionized water. The organic layer was extracted with dichloromethane, dried over anhydrous sodium sulfate, and concentrated. Purification by silica gel column chromatography (petroleum ether: dichloromethane = 1: 1) afforded $BF_2$bdk compound as a yellow solid (128.7 mg, yield: 51%). The product was further recrystallized three times from dichloromethane and n-hexane. $^1$H NMR (400 MHz, Chloroform-d, relative to $Me_4Si$/ppm) δ 8.28 (d, J = 1.7 Hz, 2H), 8.16 (dd, J = 8.1, 1.7 Hz, 2H), 7.88-7.81 (m, 4H), 7.51 (dd, J = 6.9, 1.7 Hz, 2H), 7.47-7.38 (m, 4H), 7.29 (s, 1H), 1.57 (s, 12H). $^{13}$C NMR (101 MHz, Chloroform-d) δ 182.09, 155.26, 154.51, 146.66, 137.44, 130.75, 129.53, 128.73, 127.52, 123.36, 123.05, 121.45, 120.37, 93.34, 47.29, 26.89. $^{19}$F NMR (376 MHz, Chloroform-d, 298K, relative to $CFCl_3$/ppm) δ -140.24 (20%), -140.30 (80%). FT-IR

(KBr, $cm^{-1}$): ν 3060.5, 2960.2, 2923.6, 2860.9, 1608.4, 1536.4, 1482.1, 1459.7, 1446.3, 1377.9, 1364.9, 1339.3, 1310.5, 1286.9, 1268.0, 1215.3, 1157.9, 1139.8, 1098.5, 1074.4, 1040.5, 1005.7, 964.1 908.2, 836.0, 802.2, 777.5, 758.6, 735.5, 726.3, 707.6, 565.2, 466.7, 433.3. HRMS m/z $C_{33}H_{28}BF_2O_2$ $[M+H]^+$ : calcd 505.2150, found 505.2146.

## Synthesis of PLMA Macro-CTA

In a typical experiment, LMA (5 g, 19.65 mmol), CDPA (396 mg, 0.986 mmol), AIBN (32.3 mg, 0.196 mmol), and 28.7 mL of fresh toluene were charged into a round-bottom flask, which were stirred until completely dissolved. After three freeze-pump-thaw cycles, the flask was sealed and then transferred to a constant temperature oil bath at 70 °C. After 13 h of polymerization, the reaction was quenched by liquid nitrogen and then exposed to air. When the temperature recovered to room temperature, the reaction mixture was first diluted with dichloromethane (DCM) and then added dropwise to a large amount of methanol. After the filtration, the products were collected and dried under vacuum, yielding of 4.9 g of PLMA.

## Preparation of $BF_2$bdk-PISA dispersion

In a typical reaction, $PLMA_{17}$-CTA (62.6 mg, 0.0133 mmol), 200 mg MMA (1.997 mmol) containing $BF_2$bdk with a concentration of 1 mg/mL, 108 mg of PMA (0.667 mmol) containing 0.7 mg of AIBN, and 2.3 mL of fresh n-hexane were added to a 10 mL Schlenk tube. The mixtures were stirred until completely dissolved. After three freezing-degassing-thawing cycles, the tube was sealed and transferred to a constant temperature oil bath at 70 ºC. After 24 h, the reaction was quenched with liquid nitrogen. When the temperature returned to room temperature, the $BF_2$bdk-PISA dispersion was irradiated by a 365 nm UV lamp (35 $mW/cm^2$) for around 5 s at a distance and then afterglow photographs were captured by iPhone14

cameras. After this, a small amount of the reaction mixtures was extracted use a syringe and dropped to a large amount of cold diethyl ether. The collected samples were vacuum-dried at room temperature. The remaining mixtures were stored for future use. The procedures for the synthesis of $BF_2$bdk-PISA dispersion exhibiting afterglow under different PMA/MMA feed ratios (2/8, 1/9 and 0/1) were the same to those described above.

## Synthesis of P(MMA-*co*-PMA) copolymer

P(MMA-co-PMA) was synthesized by RAFT polymerization in toluene at 70 ºC using AIBN and CDPA as the initiator and chain transfer agent, respectively. 1 g MMA, 0.5 g PMA, 1.1 mg of AIBN, 13.2 mg of CDPA and 9.4 mL fresh toluene were charged in turn to a round-bottom flask. After three freezing-degassing-thawing cycles, the flak was sealed and transferred to a constant temperature oil bath at 70 ºC. After 24 h, the reaction was quenched with liquid nitrogen. When the temperature returned to room temperature, the reaction mixtures was diluted with DCM and then dropped to a large amount of cold methanol. The collected samples were vacuum-dried at room temperature.

## Physical measurement and instrumentation

The number-average molecular weight (Mn) and molecular weight distribution of the polymers were analyzed by GPC (Waters 1515, America). The GPC system was equipped with four HR model chromatographic columns and a waters 2414 differential refractive index detector. Narrow-distribution polystyrene (PS) was used as the standard sample. THF was used as the mobile phase at a flow rate of 1.0 mL/min and a test temperature of 35 ºC. $^1$H NMR and $^{13}$C{$^1$H} NMR spectra were recorded in either $CDCl_3$ or $d_6$-DMSO on a Bruker spectrometer (AVANCE Ⅲ 400). FT-IR spectra were recorded on a Nicolet AVATAR-360 FT-IR

spectrophotomerter with a resolution of 4 $cm^{-1}$. UV-vis absorption spectra were recorded on a Techcomp UV1050 UV vis spectrophotometer. Emission spectra were recorded using Edinburgh FLS1000 fluorescence spectrometer, Hitachi FL-4700 fluorescence spectrometer. Typically, 64 scans were averaged per spectrum. Chemical shifts are expressed in ppm and are internally referenced to the residual solvent peak and tested at room temperature. Dynamic light scattering (DLS) were performed using a Zetasizer Nano ZS instrument (Malvern Instruments, UK) at a fixed scattering angle of 173°. Copolymer dispersion were dispersions were diluted in n-dodecane (0.10% w/w) prior to lights scattering analysis at 25 °C. The intensity-average diameter and polydispersity of the dispersion were calculated by cumulants analysis of the experimental correlation function using Dispersion Technology software version 6.20. Data were averaged over thirteen runs each of thirty seconds duration. The morphologies of polymer nanoparticles were observed with TEM (JEOL JEM-2100F, Japan) with an acceleration voltage of 200 kV and SEM (JEOL JSM-7800F, Japan) with an acceleration voltage of 30 kV. DSC measurements were performed using a Taber DSCQ10 Discovery series instrument operating from -50 °C to 200 °C at a rate 10 °C/min using aluminum pans and hermetic lids. The sample was heated to 200 °C and kept for 2 min at this temperature in order to eliminate the influence of thermal history, then cooled to 30 °C to record the cooling curve, and then reheated to 200 °C, all at a rate of 10 °C/min. The steady-state and delayed emission spectra were collected by Hitachi F-4700 fluorescence spectrometer equipped with chopping systems; the delayed emission spectra were obtained with a delay time of approximately 1 ms. The excited state decay profiles in millisecond to second region were collected by Hitachi F-4700 fluorescence spectrometer equipped with chopping systems. Photoluminescence

quantum yield was measured by a Hamamatsu absolute PL quantum yield measurement system based on a standard protocol.

## TD-DFT calculations

TD-DFT calculations were performed to study the photophysical properties of molecularly dispersed $BF_2$bdk in the solid state. Since the afterglow properties are originated from the excited states of molecularly dispersed $BF_2$bdk in the rigid P(MMA-co-PMA) core where intramolecular rotation and vibration are largely restricted, the ground-state geometry of $BF_2$bdk was used for all the TD-DFT calculations. The ground-state geometry of $BF_2$bdk compounds were optimized by a DFT calculation using B3LYP functional and 6-31G (d, p) basis set. The singlet excited states and triplet excited states were calculated on ORCA 5.0.4 program with B3LYP functional and def2-TZVP(-f) basis set and analyzed by Multiwfn software. Spin-orbit coupling (SOC) matrix elements between the singlet excited states and triplet excited states were calculated with spin-orbit mean-field (SOMF) methods on ORCA 5.0.4 program with B3LYP functional and def2-TZVP(-f) basis set. The obtained electronic structures were analyzed by Multiwfn software. All isosurface maps to show the electron distribution and electronic transitions were rendered by Visual Molecular Dynamics (VMD) software based on the exported files from Multiwfn.

**Table S1.** Photophysical properties of $BF_2$bdk in different solvents.

| Solvent | $\lambda_{abs}$ (nm) | ε ($10^4$ $M^{-1}cm^{-1}$) | $\lambda_{em}$ (nm) | Stokes shift ($cm^{-1}$) | $\Phi_F$ (%) |
|---|---|---|---|---|---|
| Tol | 444, 424 | 6.88, 6.12 | 467 | 1109 (23 nm) | 54.6 |
| EA | 439, 420 | 7.62, 6.86 | 467 | 1366 (28 nm) | 71.3 |
| DCM | 445, 425 | 7.14, 6.25 | 487 | 1938 (42 nm) | 73.9 |
| MeCN | 441, 422 | 7.72, 6.92 | 489 | 2226 (48 nm) | 76.8 |
| MeOH | 441, 423 | 7.56, 6.69 | 489 | 2226 (48 nm) | 73.5 |
| DMSO | 453, 434 | 7.51, 6.86 | 499 | 2035 (46 nm) | 28.7 |

Tol, EA, DCM, MeCN, MeOH and DMSO refer to toluene, ethyl acetate, dichloromethane, acetonitrile, methanol and dimethyl sulfoxide, respectively.

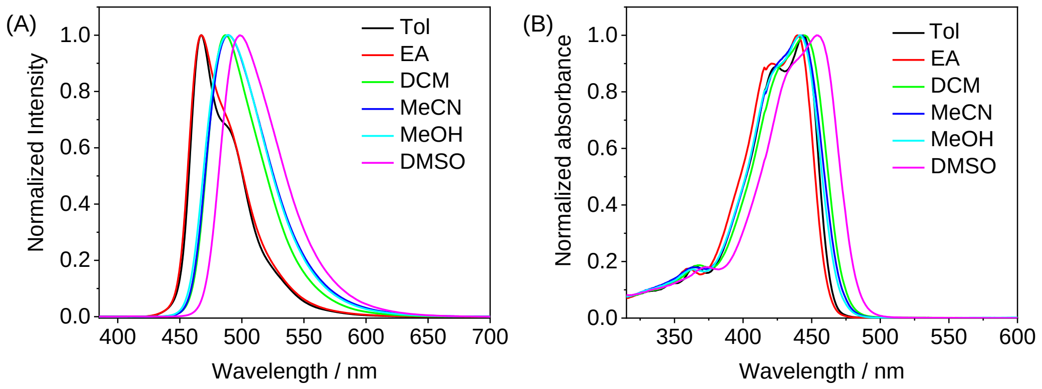


**Figure S1.** (A) UV-vis absorption spectra of $BF_2$bdk in different solvents; (B) Steady-state emission spectra of $BF_2$bdk in different solvents. Tol, EA, DCM, MeCN, MeOH and DMSO refer to toluene, ethyl acetate, dichloromethane, acetonitrile, methanol and dimethyl sulfoxide, respectively.

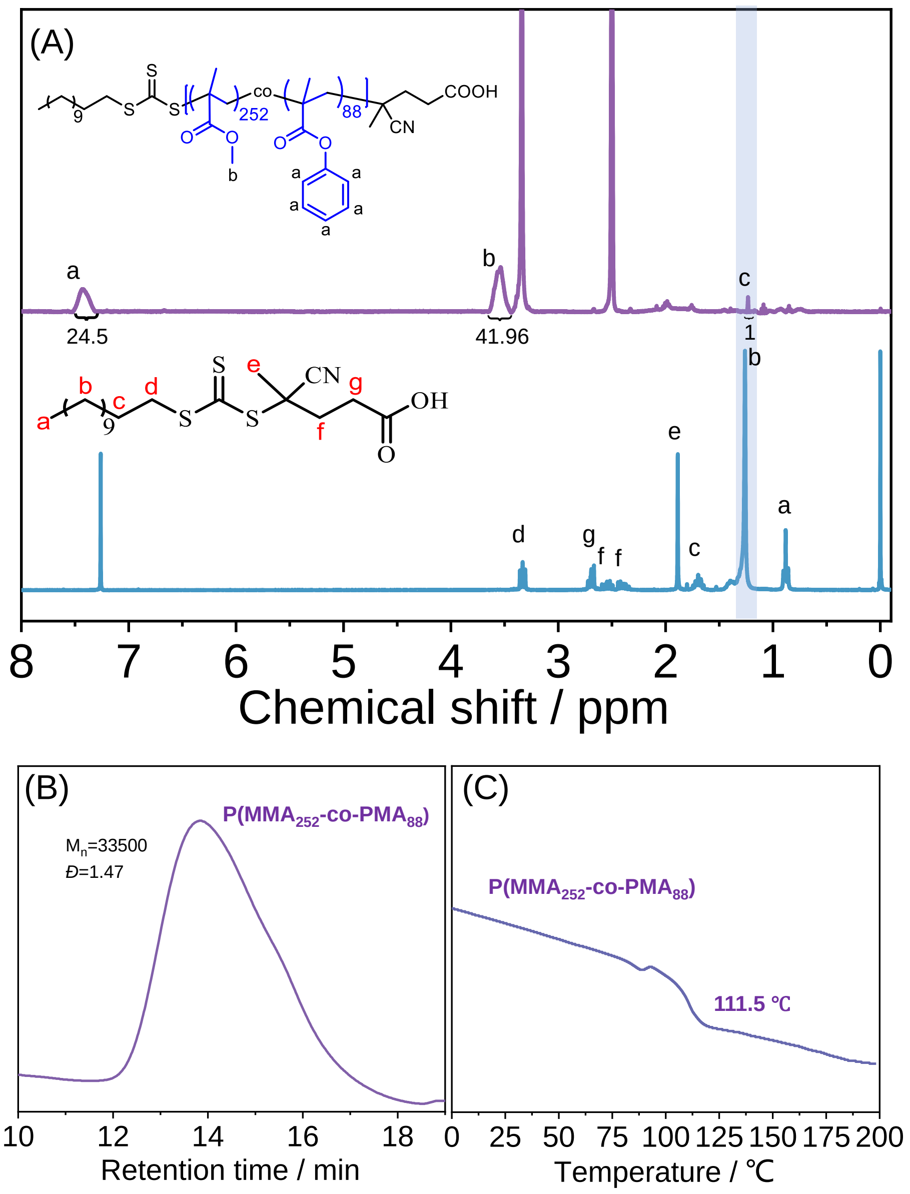


**Figure S2.** (A) $^{1}$H NMR of CDPA recorded in *d*-$CDCl_3$ and P($MMA_{252}$-*co*-$PMA_{88}$) recorded in *d*-DMSO; (B) GPC trace of P($MMA_{252}$-*co*-$PMA_{88}$); (C) DSC trace of P($MMA_{252}$-*co*-$PMA_{88}$) recorded in $N_2$ atmosphere at a heating rate of 10 ºC/min.

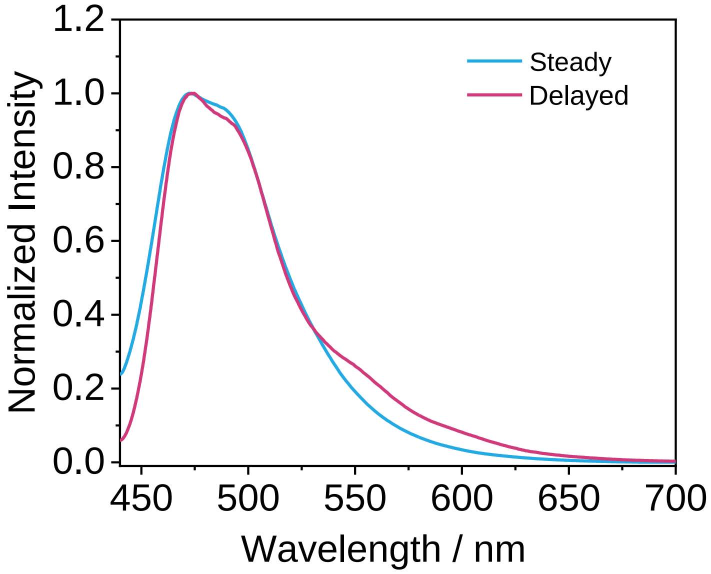


**Figure S3.** The steady-state and delayed emission spectra of $BF_2$bdk-PISA dispersions under visible light excitation monitored at 465 nm.

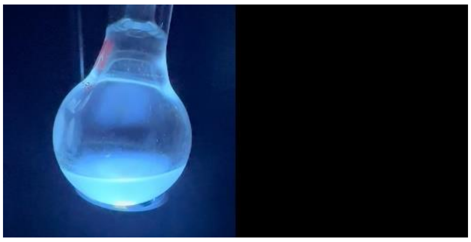

**Figure S4.** Room-temperature afterglow photographs of the PISA dispersion without $BF_2$bdk captured by iPhone14 cameras.

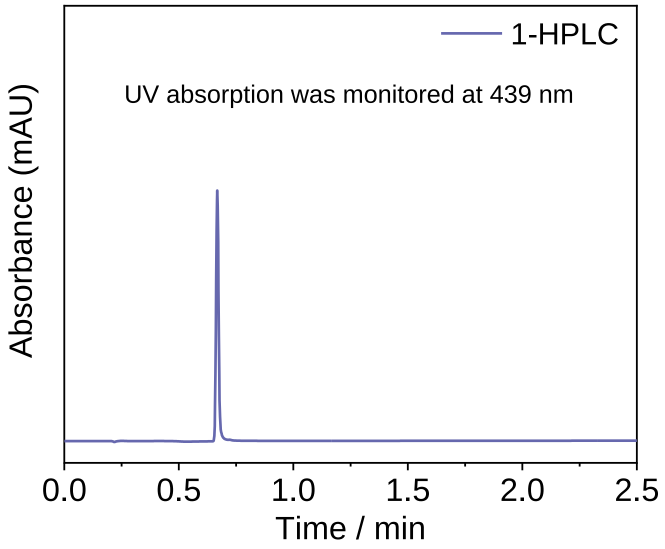


**Figure S5.** High-performance liquid chromatography (HPLC) of the purified $BF_2$bdk.

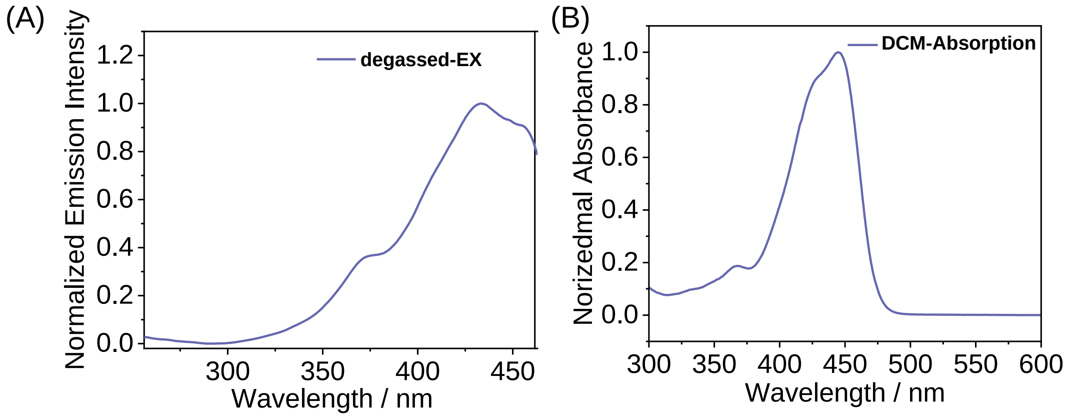


**Figure S6.** The excitation spectrum of the $BF_2$bdk-PISA dispersion and the UV-vis absorption spectrum of the isolated $BF_2$bdk dopant.

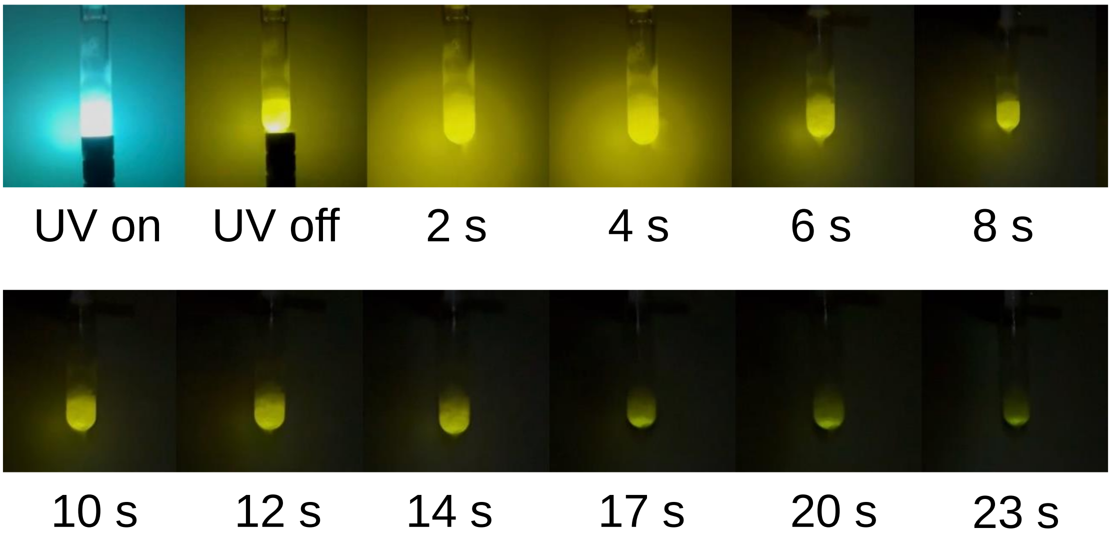


**Figure S7.** Afterglow photographs of the $BF_2$bdk-PISA dispersion at 77 K.

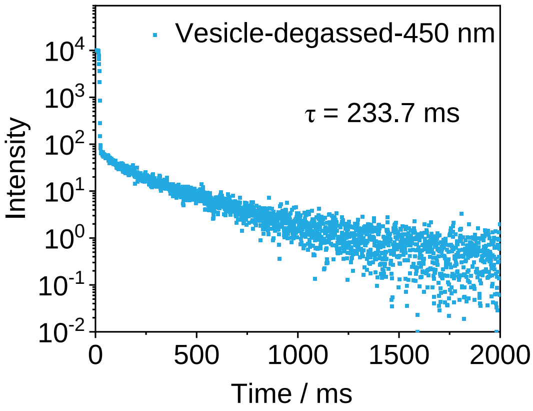


**Figure S8.** Room-temperature emission decay of the $BF_2$bdk-PISA dispersion at 450 nm.

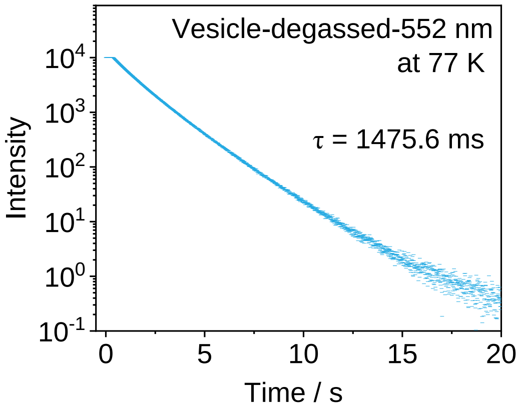


**Figure S9.** Phosphorescence emission decay of the $BF_2$bdk-PISA dispersion at 77 K.

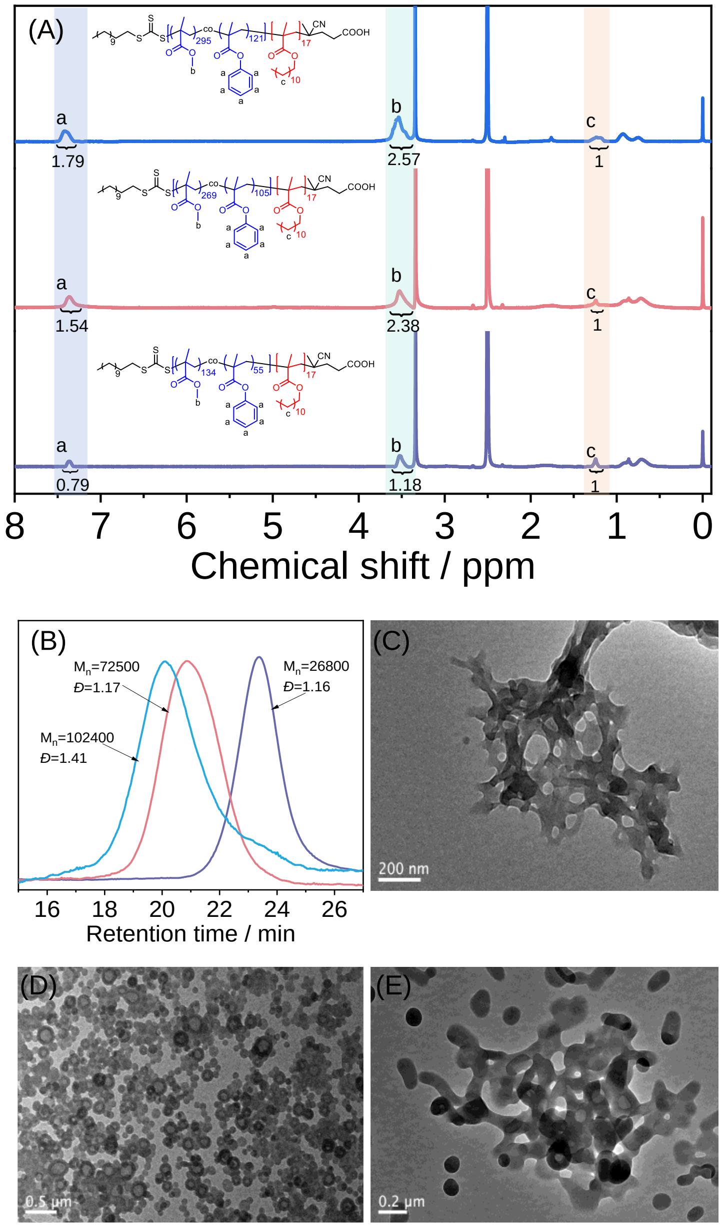


**Figure S10.** (A) $^1$H NMR spectra (in *d*-DMSO) of the $BF_2$bdk-PISA samples $PLMA_{17}$-*b*-$P(MMA_{134}$-*co*-$PMA_{55})$, $PLMA_{17}$-*b*-$P(MMA_{269}$-*co*-$PMA_{105})$ and $PLMA_{17}$-*b*-$P(MMA_{295}$-*co*-$PMA_{121})$; (B) GPC traces of the $BF_2$bdk-PISA samples $PLMA_{17}$-*b*-$P(MMA_{134}$-*co*-$PMA_{55})$, $PLMA_{17}$-*b*-$P(MMA_{269}$-*co*-$PMA_{105})$, and $PLMA_{17}$-*b*-$P(MMA_{295}$-*co*-$PMA_{121})$; (C) Represented TEM images of the $BF_2$bdk-PISA dispersion $PLMA_{17}$-$P(MMA_{134}$-*co*-$PMA_{55})$; (D) Represented TEM images of the $BF_2$bdk-PISA dispersion $PLMA_{17}$-$P(MMA_{269}$-*co*-$PMA_{105})$; (E) Represented TEM images of the $BF_2$bdk-PISA dispersion $PLMA_{17}$-$P(MMA_{295}$-*co*-$PMA_{121})$.

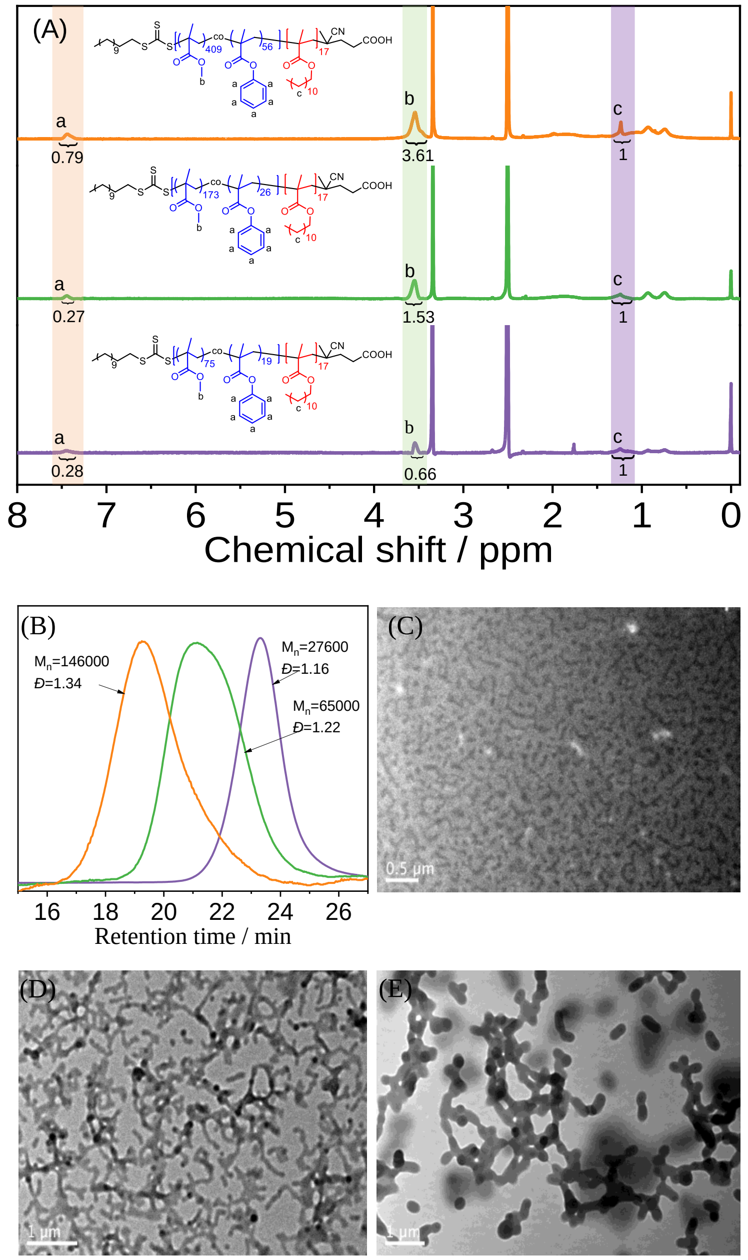


**Figure S11.** (A) $^1$H NMR spectra (in *d*-DMSO) of the $BF_2$bdk-PISA samples $PLMA_{17}$-*b*-P($MMA_{75}$-*co*-$PMA_{19}$), $PLMA_{17}$-*b*-P($MMA_{173}$-*co*-$PMA_{26}$) and $PLMA_{17}$-*b*-P($MMA_{409}$-*co*-$PMA_{56}$); (B) GPC traces of the $BF_2$bdk-PISA samples $PLMA_{17}$-*b*-P($MMA_{75}$-*co*-$PMA_{55}$), PLMA17-*b*-P($MMA_{173}$-*co*-$PMA_{26}$), and $PLMA_{17}$-*b*-P($MMA_{409}$-*co*-$PMA_{56}$); (C) Represented TEM images of the $BF_2$bdk-PISA dispersion $PLMA_{17}$-*b*-P($MMA_{75}$-*co*-$PMA_{19}$); (D) Represented TEM images of the $BF_2$bdk-PISA dispersion $PLMA_{17}$-*b*-P($MMA_{173}$-*co*-$PMA_{26}$); (E) Represented TEM images of the $BF_2$bdk-PISA dispersion $PLMA_{17}$-*b*-P($MMA_{409}$-*co*-$PMA_{56}$).

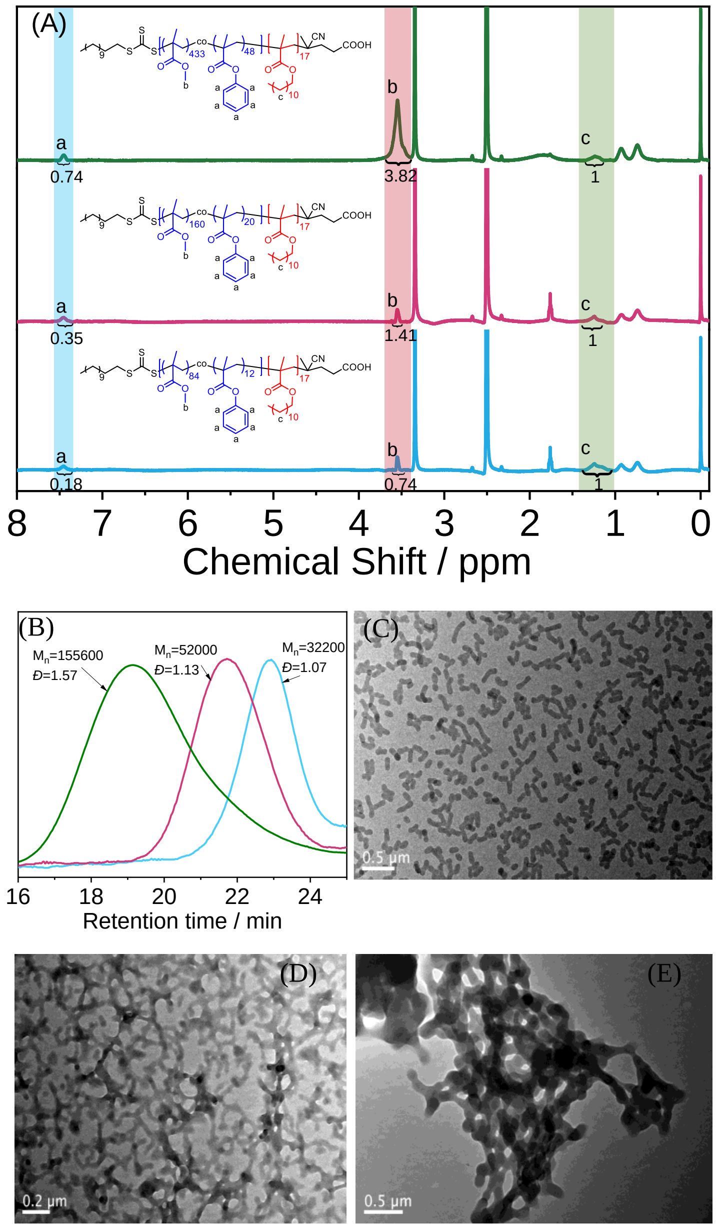


**Figure S12.** (A) $^1$H NMR spectra (in *d*-DMSO) of the $BF_2$bdk-PISA samples $PLMA_{17}$-*b*-P($MMA_{84}$-*co*-$PMA_{12}$), PLMA17-*b*-P($MMA_{160}$-*co*-$PMA_{20}$) and $PLMA_{17}$-*b*-P($MMA_{433}$-*co*-$PMA_{48}$); (B) GPC traces of the $BF_2$bdk-PISA samples $PLMA_{17}$-*b*-P($MMA_{84}$-*co*-$PMA_{12}$), $PLMA_{17}$-*b*-P($MMA_{160}$-*co*-$PMA_{20}$), and $PLMA_{17}$-*b*-P($MMA_{433}$-*co*-$PMA_{48}$); (C) Represented TEM images of the $BF_2$bdk-PISA dispersion $PLMA_{17}$-*b*-P($MMA_{84}$-*co*-$PMA_{12}$); (D) Represented TEM images of the $BF_2$bdk-PISA dispersion $PLMA_{17}$-*b*-P($MMA_{160}$-*co*-$PMA_{20}$); (E) Represented TEM images of the $BF_2$bdk-PISA dispersion $PLMA_{17}$-*b*-P($MMA_{433}$-*co*-$PMA_{48}$).

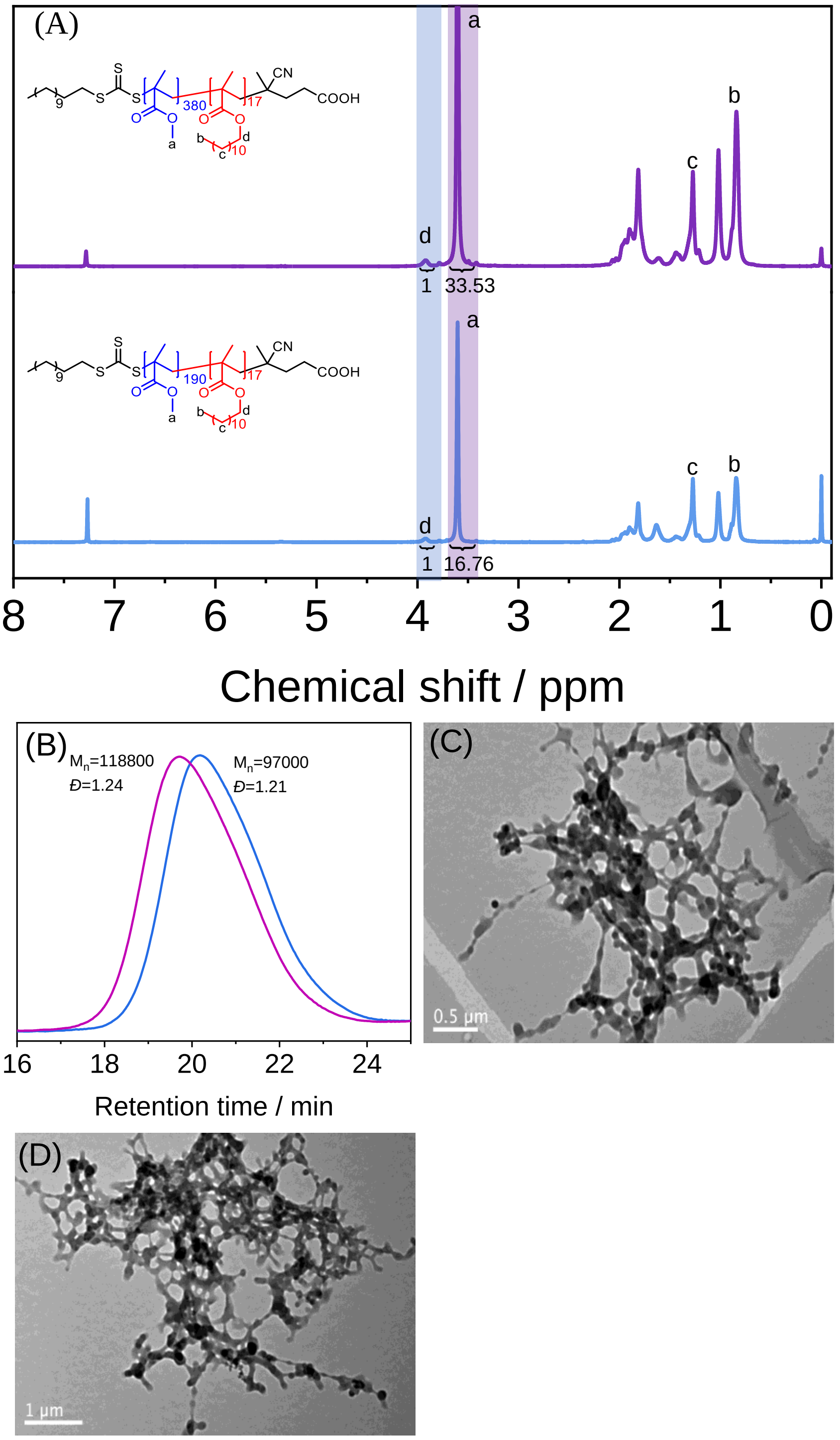


**Figure S13.** (A) $^{1}$H NMR spectra (in $CDCl_3$) of the $BF_2$bdk-PISA samples $PLMA_{17}$-*b*-$PMMA_{190}$ and $PLMA_{17}$-*b*-$PMMA_{380}$; (B) GPC traces of the $BF_2$bdk-PISA samples $PLMA_{17}$-*b*-$PMMA_{190}$ and $PLMA_{17}$-*b*-$PMMA_{380}$; (C) Represented TEM images of the $BF_2$bdk-PISA dispersion $PLMA_{17}$-$PMMA_{190}$; (D) Represented TEM images of the $BF_2$bdk-PISA dispersion $PLMA_{17}$-$PMMA_{380}$.

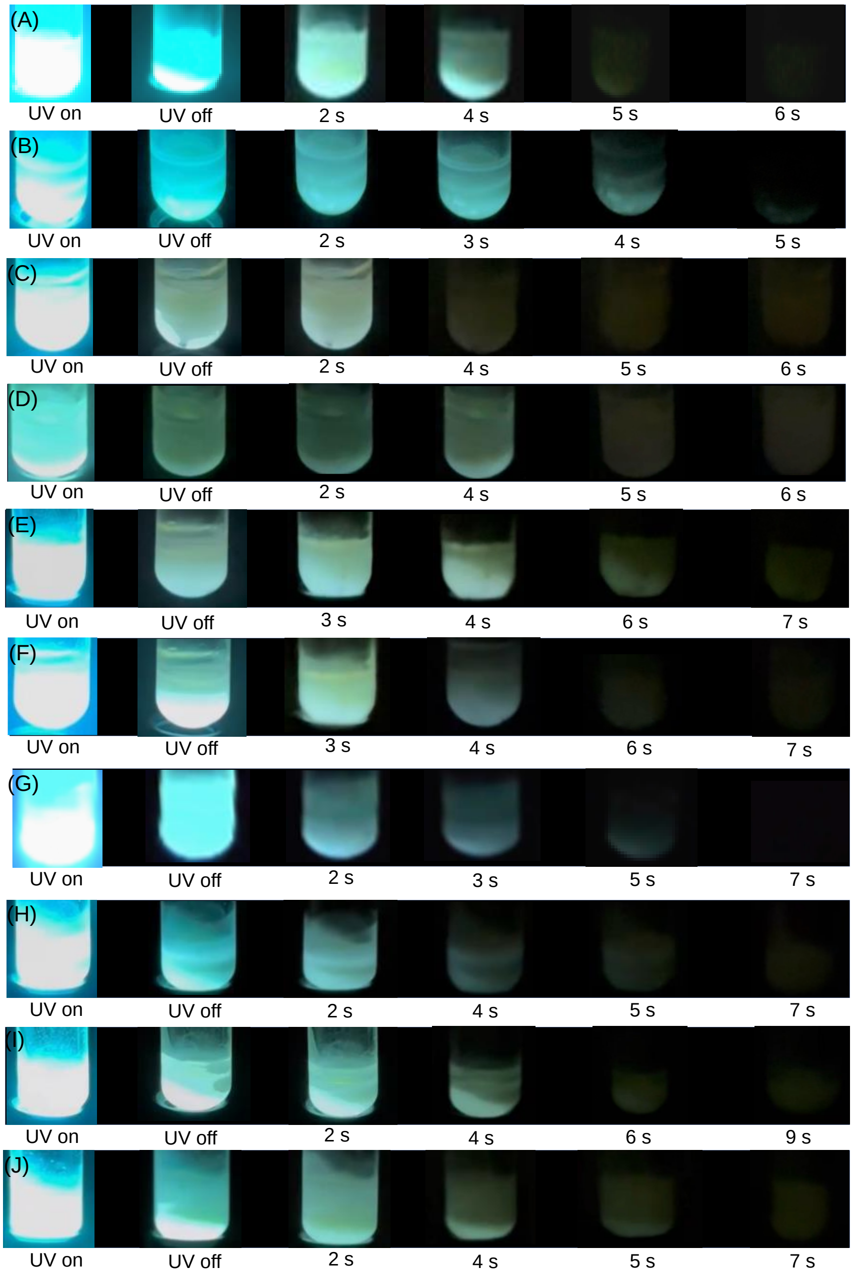


**Figure S14.** Photographs of $PLMA_{17}$-*b*-P($MMA_x$-*co*-$PMA_y$) under 365 nm UV and after ceasing the UV lamp at room temperature (A) $PLMA_{17}$-*b*-P($MMA_{134}$-*co*-$PMA_{55}$); (B) $PLMA_{17}$-*b*-P($MMA_{295}$-*co*-$PMA1_{21}$); (C) $PLMA_{17}$-*b*-P($MMA_{75}$-*co*-$PMA_{55}$); (D) $PLMA_{17}$-*b*-P($MMA_{173}$-*co*-$PMA_{26}$); (E) $PLMA_{17}$-*b*-P($MMA_{409}$-*co*-$PMA_{56}$); (F) $PLMA_{17}$-*b*-P($MMA_{84}$-*co*-$PMA_{12}$); (G) $PLMA_{17}$-*b*-P($MMA_{160}$-*co*-$PMA_{20}$); (H) $PLMA_{17}$-*b*-P($MMA_{433}$-*co*-$PMA_{48}$); (I) $PLMA_{17}$-*b*-$PMMA_{190}$; (J) $PLMA_{17}$-*b*-$PMMA_{380}$.

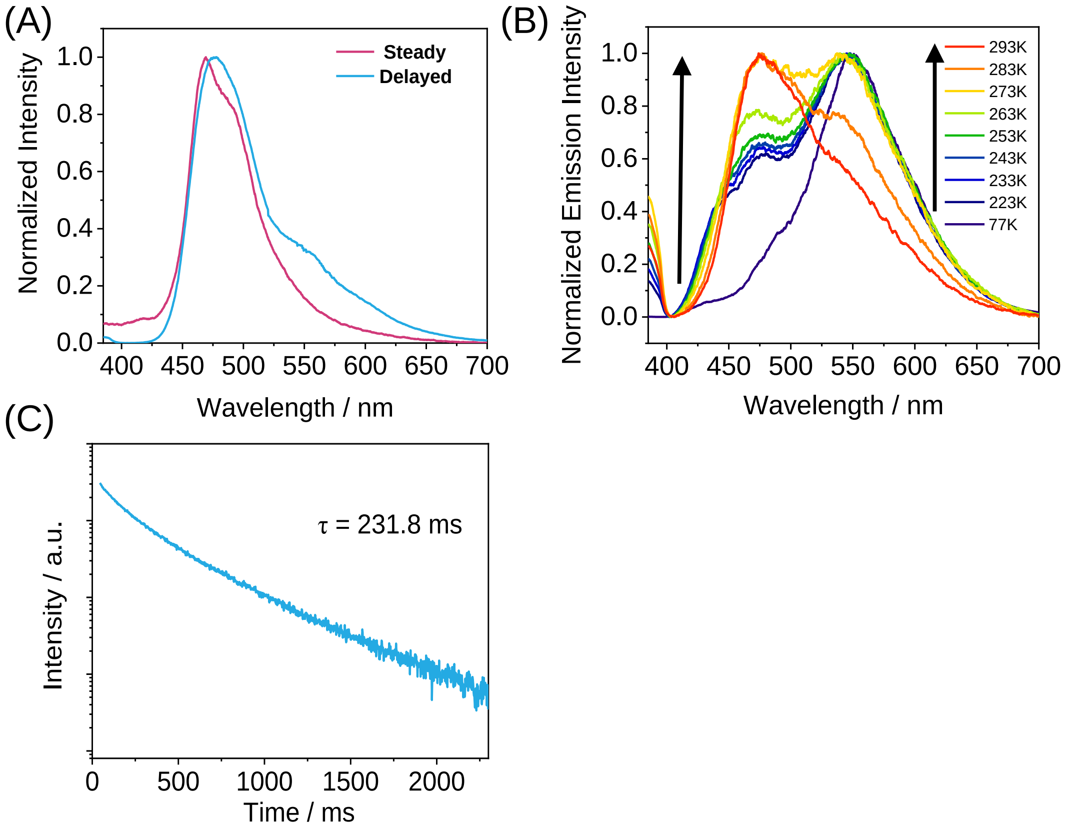


**Figure S15.** (A) Steady-state and delayed emission spectra of the $BF_2$bdk-PISA dispersion PLMA$_{17}$-*b*-P(MMA$_{80}$-*co*-PMA$_{20}$) at room temperature; (B) Temperature-dependent delayed emission spectra (1 ms delay) of the $BF_2$bdk-PISA dispersion PLMA$_{17}$-*b*-P(MMA$_{80}$-*co*-PMA$_{20}$); (C) Room-temperature emission decay of the $BF_2$bdk-PISA dispersion PLMA$_{17}$-*b*-P(MMA$_{80}$-*co*-PMA$_{20}$) monitored at 475 nm.

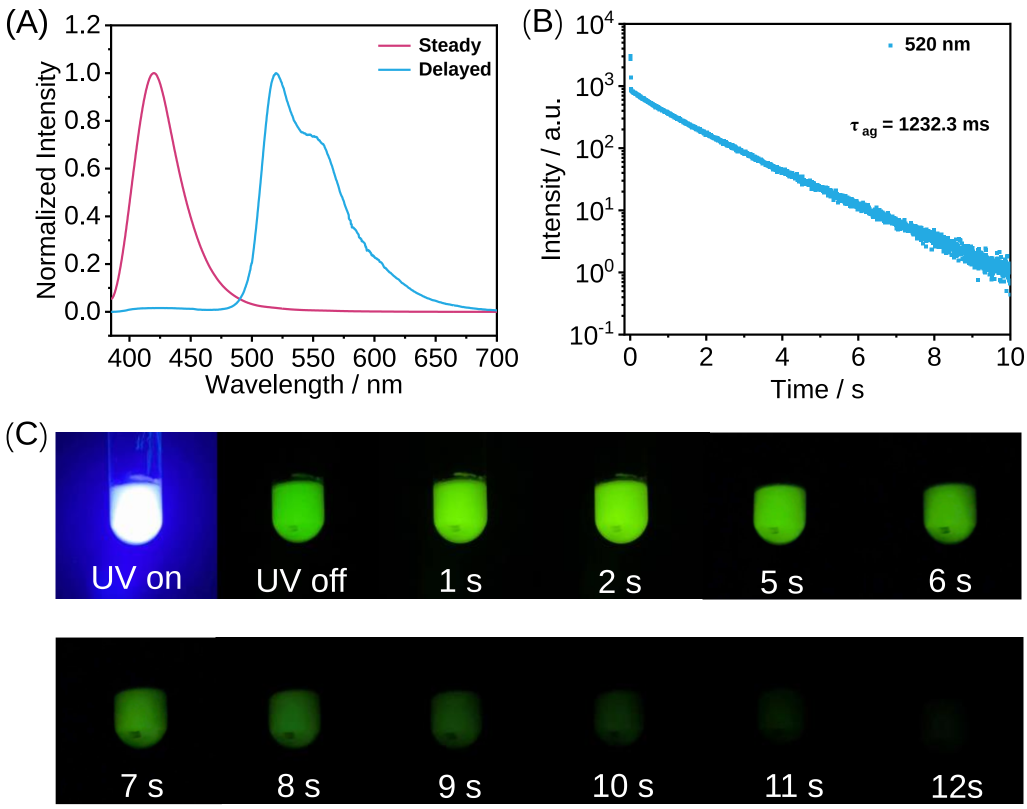


**Figure S16.** (A) Room-temperature steady-state and delayed emission spectra of the PISA dispersion formed by donor-acceptor type $BF_2$bdk (4-(9,9-dimethyl-9H-fluoren-2-yl)-2,2-difluoro-6-methyl-2H-1,3$\lambda^3$,2$\lambda^4$-dioxaborinine, that is fluorene$BF_2$ in Adv. Opt. Mater. 2021, 2100353) and $PLMA_{17}$-*b*-$PMMA_{200}$; (B) Room-temperature emission decay of the PISA dispersion formed by donor-acceptor type $BF_2$bdk and $PLMA_{17}$-*b*-$PMMA_{200}$ monitored at 520 nm. (C) Photographs of the PISA dispersion formed by donor-acceptor type $BF_2$bdk and $PLMA_{17}$-*b*-$PMMA_{200}$ under 365 nm UV and after ceasing the UV lamp at room temperature.

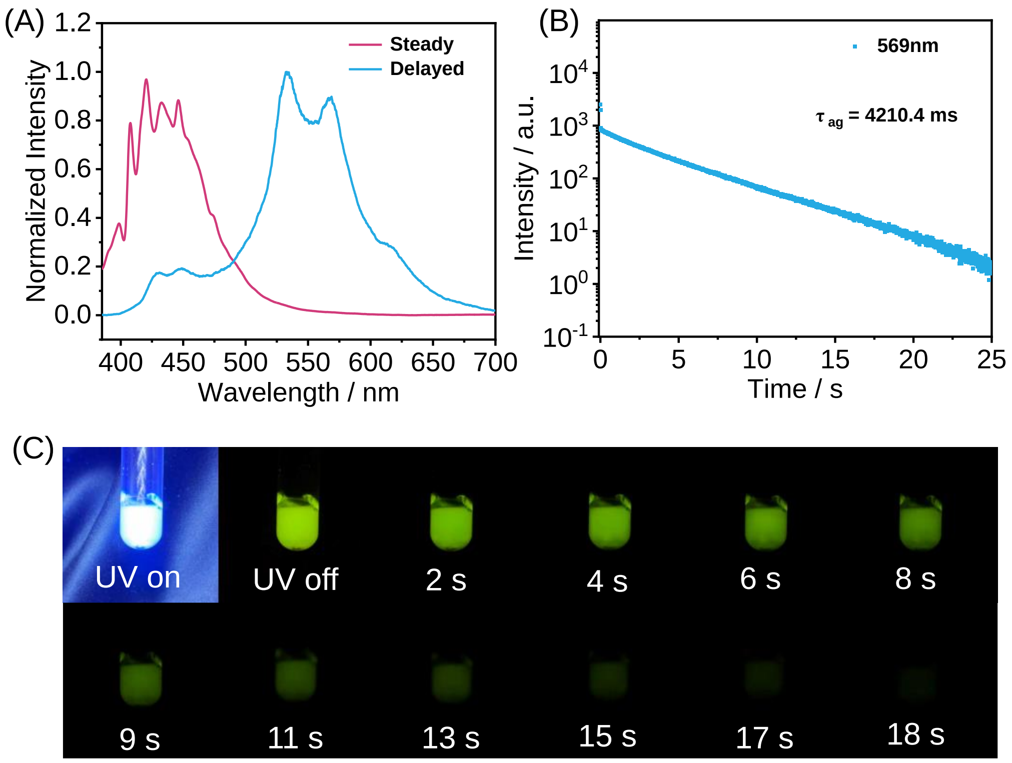


**Figure S17.** (A) Room-temperature steady-state and delayed emission spectra of the PISA dispersion formed by coronene and $PLMA_{17}$-*b*-$PMMA_{200}$; (B) Room-temperature emission decay of the coronene-PISA dispersion monitored at 569 nm. (C) Photographs of the coronene-PISA dispersion under 365 nm UV and after ceasing the UV lamp at room temperature.

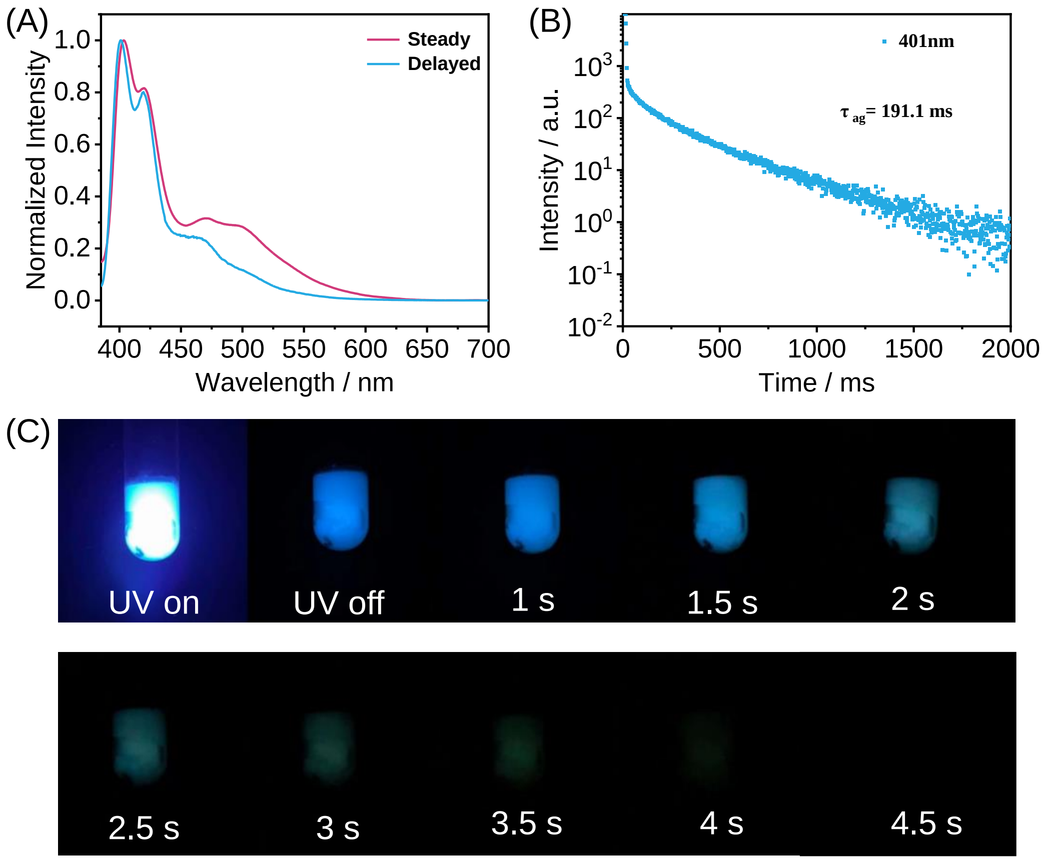


**Figure S18.** (A) Room-temperature steady-state and delayed emission spectra of the PISA dispersion formed by 10-phenylacridone and $PLMA_{17}$-*b*-$PMMA_{200}$; (B) Room-temperature emission decay of the 10-phenylacridone-PISA dispersion monitored at 401 nm. (C) Photographs of 10-phenylacridone-PISA dispersion under 365 nm UV and after ceasing the UV lamp at room temperature.

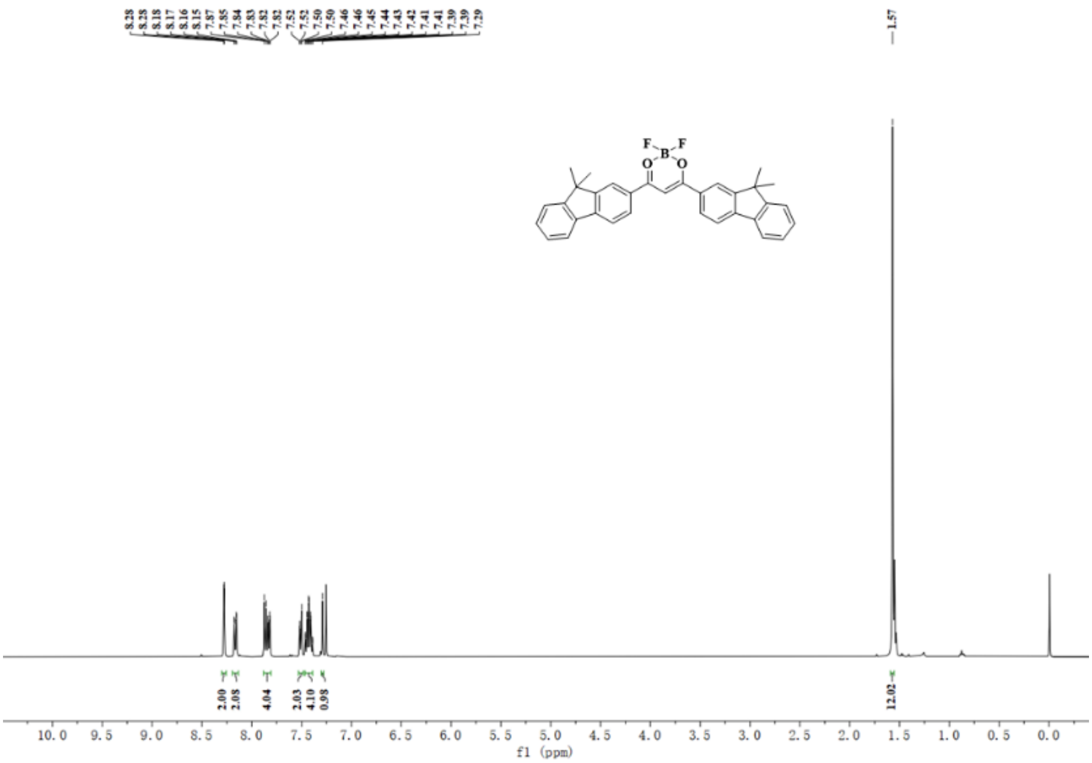


**Figure S19.** $^{1}H$ NMR spectrum of $BF_2$bdk (400 MHz, $CDCl_3$, 298 K).

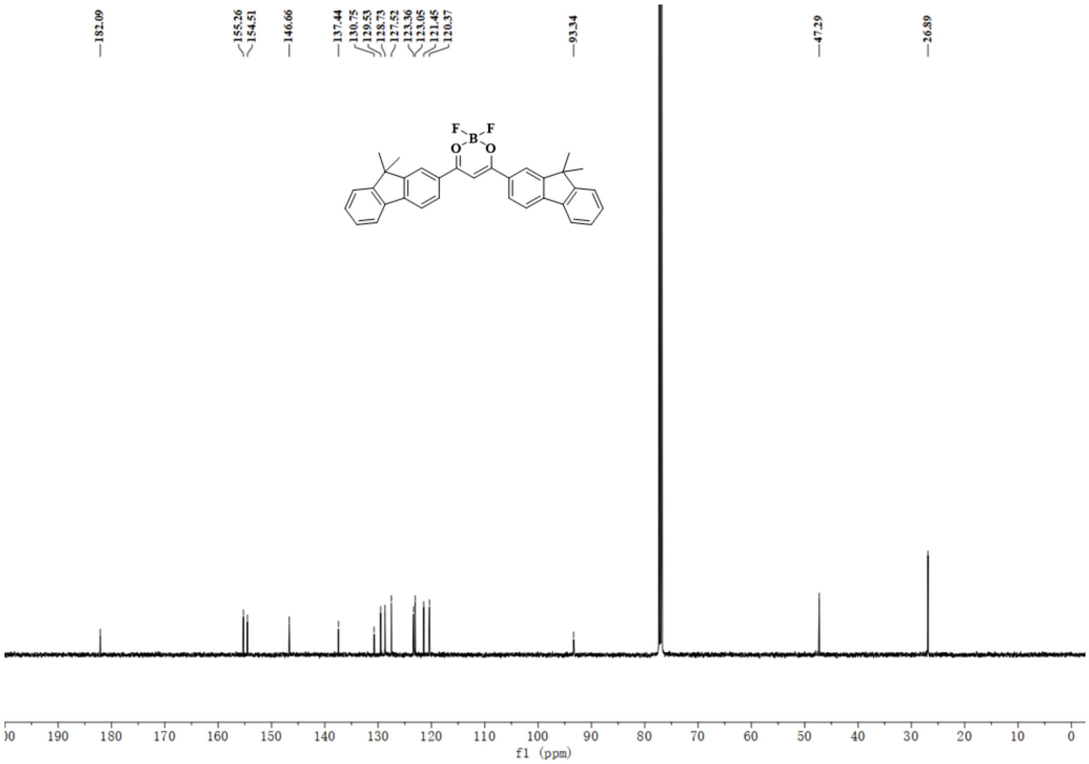


**Figure S20.** $^{13}C$ NMR spectrum of $BF_2$bdk (101 MHz, Chloroform-d).

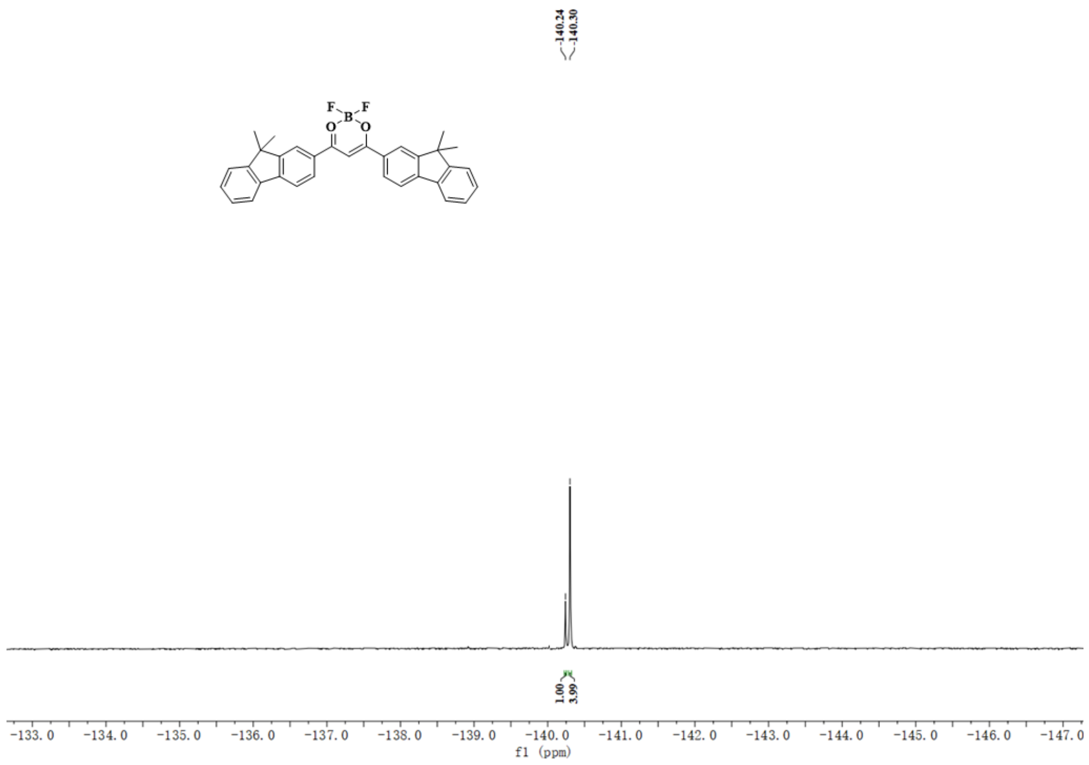


**Figure S21.** $^{19}F$ NMR spectrum of $BF_2bdk$ (376 MHz, Chloroform-d, 298K, relative to $CFCl_3$ / ppm).

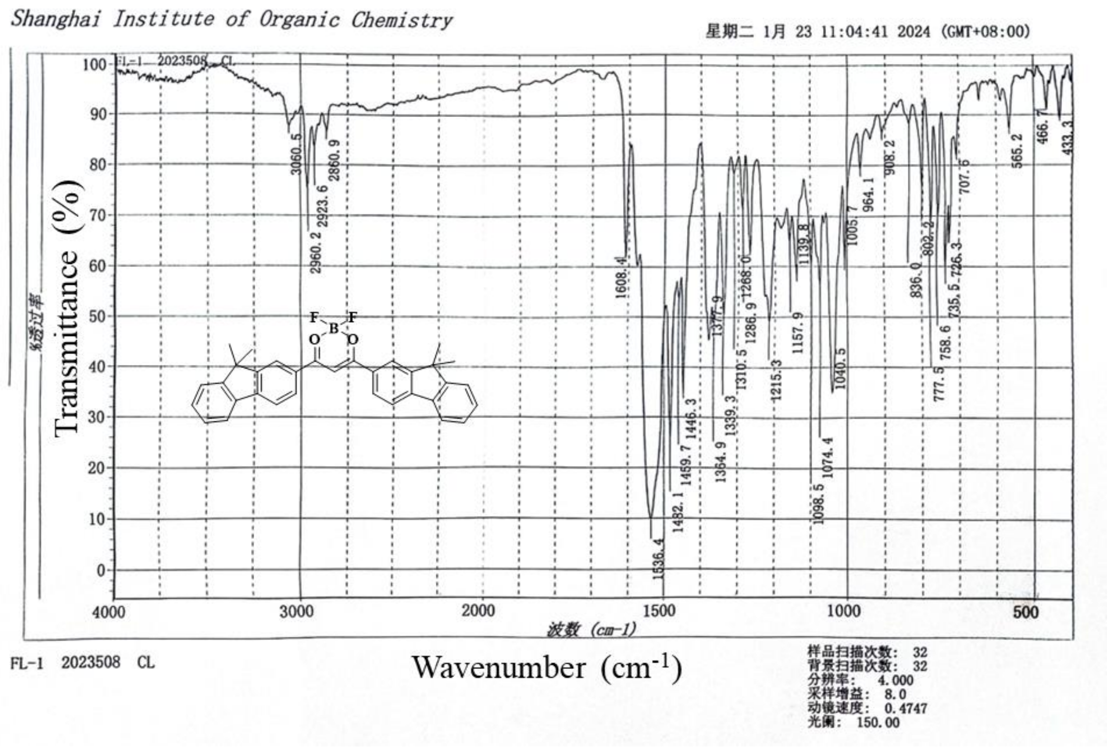


**Figure S22.** FT-IR spectrum of $BF_2bdk$ (KBr, $cm^{-1}$).

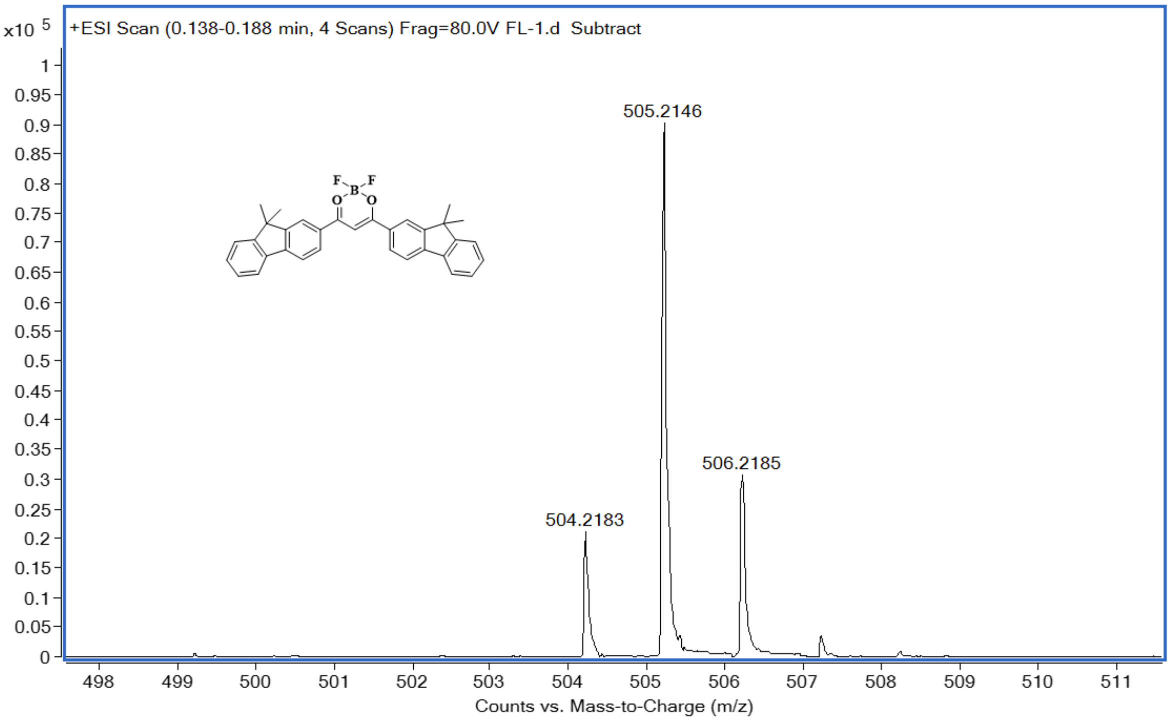


**Figure S23.** HRMS spectrum of $BF_2$bdk.

## References

[1] Y. K. C. G. Moad, A. Postma, E. Rizzardo, S.H. Thang, Advances in RAFT Polymerization: The Synthesis of Polymers with Defined End-Ggroups. *Polymer* **2005**, *46*, 8458-8468. https://doi.org/10.1016/j.polymer.2004.12.061.
